# Virulence Characteristics and Distribution of the Pathogen *Listeria* *ivanovii* in the Environment and in Food

**DOI:** 10.3390/microorganisms10081679

**Published:** 2022-08-20

**Authors:** Franca Rossi, Valerio Giaccone, Giampaolo Colavita, Carmela Amadoro, Francesco Pomilio, Paolo Catellani

**Affiliations:** 1 Istituto Zooprofilattico Sperimentale dell’Abruzzo e Molise (IZSAM) “G. Caporale”, Via Campo Boario, 64100 Teramo, Italy; 2 Dipartimento di Medicina Animale, Produzioni e Salute, Università degli Studi di Padova, Viale dell’Università, 16, 35020 Legnaro, Italy; 3 Dipartimento di Medicina e Scienze della Salute “V. Tiberio”, Università degli Studi del Molise, Via De Sanctis, 86100 Campobasso, Italy

**Keywords:** *Listeria ivanovii*, pathogenicity, virulence characters, environmental distribution, occurrence in food

## Abstract

*Listeria ivanovii* and *L. monocytogenes*, are the only pathogenic species of the genus *Listeria* and share many virulence factors and mechanisms of pathogenicity. *L. ivanovii* shows host tropism towards small ruminants and rodents and much lower virulence for humans compared to *L. monocytogenes*. However, severe infections caused by *L. ivanovii*, resulting in bacteremia, abortion and stillbirth, occasionally occurred in immunocompromised persons and in pregnant women, while in immunocompetent hosts *L. ivanovii* can cause gastroenteritis. In this review, the updated knowledge on virulence aspects and distribution of *L. ivanovii* in the environment and in food is summarized. Recent research on its virulence characters at genome level gave indications on how pathogenicity evolved in this bacterial species. As for *L. monocytogenes*, *L. ivanovii* infections occurred after the ingestion of contaminated food, so an overview of reports regarding its distribution in food products was carried out to obtain indications on the categories of foods exposed to contamination by *L. ivanovii*. It was found that a wide variety of food products can be a source of this microorganism and that, like *L. monocytogenes*, *L. ivanovii* is able to persist in the food production environment. Studies on its ability to grow in enrichment and isolation media suggested that its occurrence in nature might be underestimated. Moreover, virulence varies among strains for differences in virulence character regulation, presence/absence of genetic regions and the possible instability of a *Listeria* pathogenicity genomic island, LIPI-2, which is unique to *L. ivanovii*. We can conclude that *L. ivanovii*, as a possible pathogen for animals and humans, requires more focused investigations regarding its occurrence in the environment and in food and on intra-species variability of pathogenic potential.

## 1. Introduction

The genus *Listeria* comprises Gram-positive facultative-anaerobic bacilli ubiquitous in the environment from which they come in contact with food and feed and can be ingested by human and animals. Currently, 26 bacterial species are included in the genus *Listeria* [1] that comprises a *Listeria* sensu stricto clade, composed by *L. monocytogenes* and the closely related species *L. innocua*, *L. ivanovii*, *L. marthii*, *L. seeligeri* and *L. welshimeri*, and the *Listeria* sensu lato clade comprising genetically and phenotypically diverse species that differ from *Listeria* sensu stricto species for the lack of motility, ability to reduce nitrate, lack of acetoin production and inability to grow at 4 °C [2]. 

The *Listeria* genus comprises only two species that are pathogenic for humans and animals, *L. monocytogenes* and *L. ivanovii*. These bacteria, when ingested through contaminated food and feed, are capable of invading the intestinal epithelium and proliferate within macrophages and other cell types, causing intestinal, systemic infections, or infections in other body sites that occur when they cross the intestinal barrier. Moreover, pathogenic *Listeria* spp. can invade placental tissues, cross the maternal-fetal barrier and cause abortion or stillbirth [3].

Based on the evolutionary events inferred by whole genome comparison it was proposed that species belonging to the *Listeria* sensu stricto clade derived from a common pathogenic ancestor. Indeed, there are no indications of lateral gene transfer of the virulence gene clusters. Conversely, putative deletion breakpoints of the virulence gene clusters are found in the genomes of *L. innocua* and *L. welshimeri* that suggest the loss pathogenicity genetic determinants in two independent events [4].

Bacterial strains belonging to the species *L. ivanovii* were initially classified as *L*. *monocytogenes* serovar 5, but their status of separate species was suggested in early descriptions of these bacteria based on biochemical and serological traits [5]. The distinctness of the species *L. ivanovii* and *L. monocytogenes* was later confirmed by DNA-DNA hybridization with the SI nuclease-trichloroacetic acid method [6], and this led to the proposal of the new species *L. ivanovii* in 1984 [7]. The distinctive characteristics defined for this species were a positive synergistic hemolysis CAMP test (so named for Christie, Atkins, and Munch-Petersen) with *Rhodococcus equi* and pathogenicity for mice, but at a lethal dose 10 times higher than *L*. *monocytogenes*. Later, two subspecies of *L. ivanovii*, differentiated on the basis of enzyme allele electrophoresis profiles, were described, *L. ivanovii* subsp. *ivanovii* and *L. ivanovii* subsp. *londoniensis*, that can be discriminated on the basis of ribose fermentation, positive for the subspecies *ivanovii*, and N-acetyl-β-D-mannosamine fermentation, positive for the subspecies *londoniensis* [8]. 

Some other distinctive traits differentiate the two subspecies, namely the higher sensitivity to phage infection of *L. ivanovii* subsp. *ivanovii* attributed to the absence of a functional type II-A CRISPR-Cas system, found instead in *L. ivanovii* subsp. *londoniensis* [9], and the fact that only *L. ivanovii* subsp. *ivanovii* has been shown to cause listeriosis in human and animals [10].

*L. ivanovii* infections cause fetal death, stillbirths and premature births in ruminants, although less frequently than *L*. *monocytogenes*. Differently from *L*. *monocytogenes*, *L. ivanovii* never caused meningoencephalitis in ruminants and rarely infected humans causing primarily gastroenteritis, bacteremia in immunocompromised persons and fetal loss in pregnant women [11]. Human listeriosis caused by *L. ivanovii* still appears to be rare, with only two cases reported in the scientific literature [10,12] after those reviewed by Guillet et al., in 2010 [11]. However, this bacterial species is endowed with a complex system of virulence factors, with tendency to evolve [10,13], that constitute a pathogenic potential to be carefully evaluated and monitored.

Therefore, in this review, updates on the genetic features, infectiveness and ability to cause disease in humans and animals of *L. ivanovii* are summarized. Moreover, a worldwide analysis of *L. ivanovii* occurrence in food products was carried out in order to identify the sources that most probably can transmit this infectious agent and possible distribution trends. Despite the fact that the occurrence of *L. ivanovii* in food might be underestimated because of its limited growth capacity in the culture media used in the standard procedures applied for the isolation of all *Listeria* species [1], this analysis can constitute a contribution to an enhanced control on the ability of this bacterium to cause infections.

## 2. Virulence Characters of *L. ivanovii*

The pathogenicity of *L. ivanovii* is conferred by the presence in its genome of *Listeria* pathogenicity island 1 and 2 (LIPI-1 and LIPI-2). LIPI-1 has a counterpart in *L. monocytogenes* and comprises genes encoding the virulence gene regulator PrfA, an actin polymerization surface protein ActA, a pore forming toxin with hemolytic activity, called listeriolysin O in *L. monocytogenes* or ivanolysin O in *L. ivanovii* (genes *hly* or *llo* of *L. monocytogenes* and *ilo* of *L. ivanovii*), a metalloprotease (*mpl*) involved in the maturation of the phospholipase PlcB, and two phospholipases PlcA and PlcB [14]. The Ilo hemolysin of *L. ivanovii* and the Llo hemolysin in *L. monocytogenes*, are cholesterol-dependent pore-forming toxins (CDTX) essential for the intracellular cycle of the pathogens, since they allow the lysis of the phagocytic vacuole and the release of the bacterium in the cytoplasm of infected cells. This is the first step toward intracellular proliferation and propagation of the infection to the neighboring cells [15]. The LIPI-2 pathogenicity island is specific for *L. ivanovii* and includes genes encoding a sphingomyelinase C, SmcL, present only in this species, genes for secreted internalins (SE-inls) InlE, F, G, H, I, J, K and L and two InlB homologues, InlB1 and 2. LIPI-2 is inserted into a tRNAarg gene between genes *ysn*B and *yde* and, though it was found to be conserved in all analyzed *L. ivanovii* isolates, it was spontaneously deleted in vitro with part of the neighboring genome regions. In this region some internal rearrangements were observed among strains. In particular, the gene *inl*B2 was found to be absent in a strain of *L. ivanovii* subsp. *londoniensis*. Except for *smc*L and *inl*B1, all LIPI-1 and LIPI-2 genes are regulated by *prf*A [14]. 

The genome of *L. ivanovii* subsp. *ivanovii* PAM 55, that caused an outbreak of abortion in sheeps in Spain, was found to encode 17 soluble internalins, two paralogs of InlA and three paralogs of InlB [16]. The large internalin (LA-Inl) InlA mediates bacterial entry only into cells expressing E-cadherin, whereas the other LA-Inl, InlB, is a more versatile invasion factor that binds to different widely expressed receptors [17]. The InlB1 and InlB2 of *L. ivanovii* are similar to InlB of *L*. *monocytogenes* for the presence of GW modules that allow their attachment to the bacterial surface using lipoteichoic acid as ligand. InlB of *L. monocytogenes* mediates bacterial entry in the host cells by exploiting a host process called “polarized exocytosis” [18]. However, its role in the invasion of epithelial cells through binding to the E-cadherin receptor and stimulation of actin polymerization appeared to be secondary compared to that of InlA. The remaining eight LIPI-2-encode SE-Inls that share extensive sequence similarity with one another and with other *L. ivanovii* SE-Inls described previously, i.e., those from the *inl*DC locus [19], and to InlC of *L*. *monocytogenes* [14]. Noticeably, in studies with deletion mutants Dominguez-Bernal et al. [14] demonstrated for the first time that internalins encoded by LIPI-2 favor apoptosis in infected host cells. 

The phospholipase SmlC is responsible for the bizonal hemolysis and a shovel-shaped cooperative lytic “CAMP-like” reaction with *Rhodococcus equi* which is exploited for phenotypic identification of the species *L. ivanovii*. It was suggested that SmlC has a role in host tropism since it lyses sheep erythrocytes but not horse erythrocytes that have significantly lower amounts of sphingomyelin [20]. An important mechanism of pathogenesis of *L*. *monocytogenes* is the ability to cause the polymerization of host cell actin with formation of actin tails attached to one pole of the bacterium. These actin filaments grow and propel the bacterium toward the cell membrane. Here protrusions containing *Listeria* cells are formed and are incorporated by adjacent cells giving rise to a new intracellular infection cycle. Actin polymerization is catalyzed by the listerial protein ActA [17]. The gene *iact*A of *L. ivanovii* is homologous to the *act*A gene of *L. monocytogenes* and was cloned and characterized from *L. ivanovii* CLIP257. This gene encodes a protein of 1044 amino acids that shares a similar structure with ActA, though it appears distantly related. When expressed in an *L*. *monocytogenes act*A deletion mutant, this gene restored actin polymerization [21]. However, *L. ivanovii* was reported to induce intracellular actin polymerization to a lesser extent than *L. monocytogenes*. Moreover, though *L. ivanovii* is capable of cell-to-cell diffusion, it kills infected cells less efficiently than *L*. *monocytogenes* [22]. The LIPI-2 region was suggested to represent a ‘‘hot spot’’ of genome evolution in *Listeria* spp., and it was probably acquired by transduction by *L. ivanovii* [23]. 

Beye et al. [10] analyzed the virulence genes encoded by the strain *L. ivanovii* G770, isolated from a patient with aortic prosthesis infection. This strain possessed all six genes of the LIPI-1 cluster but showed sequence variation in the genes *ilo* and *act*A compared to other strains of *L. ivanovii* subsp. *ivanovii*. However, the domains involved in virulence were conserved. Therefore, the authors did not attribute the increased virulence of strain G770 to sequence variation in LIPI-1 but rather to the presence of a type I restriction-modification system described for the first time in that *L. ivanovii* strain. The restriction-modification systems effectively allow discrimination of self and non-self DNA in bacteria and protect bacteria against phages, plasmids and transposons. In addition, type I restriction-modification systems play roles in host defence, virulence, control of the evolution speed and capacity to colonize new habitats. In the genome of *L. ivanovii* G770 other strain-specific genes found were those encoding a membrane protein, a S-transferase, a DNA helicase, an acquired *van*Z gene, a few hypothetical proteins, a DNA metylase, a DNA mismatch repair protein, a F-box/FBD/LRR protein of unknown function and an Acetyl-CoA synthetase. In the study regarding *L. ivanovii* G770 it was also observed that strains differ in the number of Clustered Regularly Interspaced Short Palindromic Repeats (CRISPR) regions that varies between one and three. Among the genes responsible for virulence the stress survival islet 1 (SS1) was also considered.

In *Listeria* spp. adaptive genes which may play a vital role in the response to different environmental stressors are found also on extra-chromosomal replicons. The G5 group of these replicons is the most divergent, and two plasmids from this group, pLIS46 (MZ147617) and pLIS47 (MZ147618) were found to coexist in *L. ivanovii* strain Sr19, suggesting that also in this species these genetic elements can promote adaptation to different environments [24].

Gan et al. [13] analyzed the virulence characteristics of *L. ivanovii* subsp. *ivanovii* strains isolated from intestinal contents of wild rodents in China and belonging to the sequence types (STs) 1 and 2. The multilocus sequence typing (MLST) scheme used for *L. ivanovii* was adopted by Cao et al. [25] and was the same as for *L. monocytogenes*. It included the housekeeping genes *abc*Z, *bgl*A, *cat*, *dap*E, *dat*, *ldh*, and *lhk*A, with a single nucleotide difference considered as a distinct allele. The ST of each isolate was defined by the combination of numbers corresponding to the allele’s loci in the local database. The *L. ivanovii* strains PAM 55 was a representative of ST1. *L. ivanovii* strains were assigned to 11 STs and divided into two lineages. Lineage I corresponds to *L. ivanovii* subsp. *ivanovii* and comprises ST1, ST2, ST4, ST7, ST8, and ST9, whereas lineage II corresponds to *L. ivanovii* subsp. *londoniensis* and comprises ST5, ST6, ST10, and ST11. 

Among the *L. ivanovii* subsp. *ivanovii* isolates, ST1 and ST2 predominated [13]. ST1 comprised more virulent strains from *Mus pahari*, *Apodemus chevrieri*, *A. draco* and *Niviventer confucianus* collected in Tibet and in Yunnan province and represented 8.62% of the isolates, while ST2 comprised 86.21% of the isolates. The ST2 isolates originated from *Ochotona curzoniae* (pikas) and *Marmota himalayana* of the Qinghai province. One ST1 and one ST2 representatives were compared genetically and phenotypically. The ST1 strain showed a cloudy growth and swarming motility in semisolid stab similarly to *L. ivanovii* PAM 55 at 25 °C, while the ST2 strain appeared non motile. Indeed, a flagellum was observed in the ST1 strain, but not in ST2 strain, with a transmission electron microscope. Notably, in ST2 strains, a premature stop codon (PMSC) was identified in the regulator gene *gma*R, that regulates expression of flagellar genes, and 24 motility related genes have non-synonymous mutations and/or indels compared with strain PAM 55. The relative expression of *gma*R and *fla*A in the ST2 strain LIV047 were significantly lower than that in PAM 55 and the ST1 strain. In the ST2 strain, deletions and several non-synonymous mutations were identified in *act*A, *inl*B2 and *agr*C. Moreover, this strain showed lower expression levels of the *plc*A gene than ST1 strains that corresponded to the inability to generate a white halo in agar medium containing phosphatidylcholine. In addition, the ST2 strain utilized glycerol, ethanolamine, amino acids, and peptides less efficiently than the ST1 strain. All these genetic differences can account for the lower virulence observed for the ST2 strain in mice. 

## 3. *L. ivanovii* Invasiveness

The cell invasion capacity of *L. ivanovii* was demonstrated for different host cell types of human and animal origin. 

Guillet et al. [11] reported that the *L. ivanovii* isolates from a patient with gastroenteritis and bacteremia were hyperinvasive in Madin-Darby bovine kidney (MDBK) cells, even more than *L. monocytogenes*, and less invasive in HeLa cells. They also performed invasion assays with cells expressing or not human E-cadherin that did not show substantial differences in invasiveness for the two different cell types, suggesting that *L. ivanovii* InlA does not interact with E-cadherin.

Alvarez-Ordóñez et al. [26] showed, by cell invasion assays, that the majority of *L. ivanovii* strains had comparable ability to invade CaCo-2 epithelial cells with *L. monocytogenes* EGDe, while four isolates had even higher invasion efficiencies.

Ammendolia et al. [27] demonstrated that *L. ivanovii* is able to adhere to human amniotic cells, invade the cytoplasm, lyse the phagosome, synthetize actin tails and spread among adjacent cells as efficiency as *L. monocytogenes*. However, *L. ivanovii* showed a lower survival capacity in the host cell cytoplasm compared to *L. monocytogenes*. 

Rocha et al. [28], using *L. ivanovii* type strain ATCC 19119, demonstrated for the first time the susceptibility of bovine trophoblastic cells to *L. monocytogenes* and *L. ivanovii*, that can explain the abortions and reproductive failures caused by *L. ivanovii* in cattle. 

Gan et al. [13] reported high invasion ability, cytotoxicity and intracellular growth in CaCo-2 and MDBK cells for a *L. ivanovii* ST1 strain. Growth in cells appeared from 3 to 6 h post-infection in both cell types. This strain caused a remarkable weight loss and injuries in liver and spleen in an intraperitoneal infection trial in mice. 

In a study carried out in vivo in mice the invasiveness of *L. ivanovii* appeared to be much lower than that of *L. monocytogenes*. In mice intravenously inoculated with 5 × 10^5^ CFU of *L. ivanovii* PAM 55, about 88% of the bacteria invaded liver and decreased gradually. Lesions were few but large and consisted of layers of necrotic hepatocytes and lymphocytes. The load of *L. ivanovii* in the spleen and in the lung decreased to below the detection limit after 3 days post infection (dpi) and no lesions were observed in spleens, thus showing a limited ability of the strain to maintain infection. In the lung collapsed alveoli accompanied with lymphocytes appeared. After intranasal inoculation, *L. ivanovii* was localized in the lung, where it remained at high loads until 5 dpi and then dropped sharply, while liver and spleen were invaded very little. Tissue damage of the lungs was severe but with lesions densely packed, indicating a limited ability of *L. ivanovii* to enlarge the infection foci. The hepatic lesions were small and splenic necrosis was hardly observed [29].

In an experimental infection of broiler chicken with 1.5 *×* 10^8^ CFU of *L. ivanovii* UNCSM–042, isolated in Ukraine, post-mortem examination after 23 dpi allowed to observe an enlargement of the spleen, an overfilled gallbladder, congestive hyperemia of the internal organs, and hyperplasia of the intestinal vessels. However, the growth level of the infected animals was not affected compared to the controls [30]. Based on the available evidences, limited invasiveness in vivo can account for the rare occurrence of *L. ivanovii* infections.

## 4. *L. ivanovii* Persistence and Tolerance to Harsh Conditions

Environmental persistence of *Listeria* spp. is determined by the capacity of these bacteria to form biofilms. Nyenje et al. [31] investigated the biofilm forming capacity of *L. ivanovii* strains and observed that 88% of the strains were able to form biofilm at 25 °C with four biofilm phenotypes. This indicated the ability of the *L. ivanov*ii species to adhere at room temperature to surfaces and utensils not properly cleaned, from which it can contaminate food. A high persistence capacity of *L. ivanovii* was indeed reported for a cheese production plant where the same pulsed field gel electrophoresis (PFGE) *Asc*I and *Apa*I pulsotype of *L. ivanovii* was isolated over a six-month period [32]. 

Determinants conferring resistance to cadmium and arsenic are widely distributed among *Listeria* species and an association was observed between resistance to cadmium and resistance to benzalkonium chloride, a sanitizer commonly used in food industries. To date, six cadmium efflux systems have been described in *Listeria* spp. that are located on transposons inserted in plasmids or within integrative conjugative elements (ICE) in the chromosome. It was observed that the presence of some *cad*AC resistance cassettes in *Listeria* spp. can influence virulence and biofilm formation. Among the cadmium resistance determinants described to date, *cad*A6b was found to be encoded by the *L. ivanovii* plasmid pLIS6, the first plasmid characterized for this species, in which the *cad*A6b cassette was probably introduced via a 6-kb non-composite transposon [33]. Resistance of *L. ivanovii* to benzalkonium chloride was directly investigated in a study regarding the distribution of the Tn6188 transposon of *L. monocytogenes*, encoding the multidrug resistance transporter QacH, in other *Listeria* species, and it was found that the ten *L. ivanovii* strains considered did not harbor this transposon [34].

In another study it was observed that two *L. ivanovii* strains isolated from postharvest sources in fresh produce processing could adapt to levels of benzalkonium chloride 3-fold higher than non-adapted wild types for the arising of nonsense mutations in the *fep*R regulator gene of the *fep*RA operon, which encodes the efflux pump FepA [35].

The tolerance to low pH values was analyzed in relation to the cell invasion capacity and at different levels of iron availability. *L. ivanovii* subsp. *ivanovii* ATCC 19119 was not able to grow at pH 5.1 and exposure to this pH did not trigger an acid tolerance response (ATR) for adaptation to lower pH values. Indeed, the bacterium was rapidly killed at pH 3.5. Acid-adapted cells showed a higher percentage of internalization in CaCo-2 cells when iron was added to the culture medium. Iron depletion enhanced the capacity of the bacterium to invade amniotic cells, regardless of acid adaptation or not [36]. 

## 5. Environmental Distribution of *L. ivanovii*

Investigations on the distribution of *L. ivanovii* in the environment mainly regarded its presence in animals and it was isolated from mastitis cases in cattle and buffalo [37], from aborted goats (7.5%), mastitic goats (5.6%) and healthy goats (14.5%) [38]. 

Studies carried out in China indicated that wild rodents could represent a reservoir of bacteria belonging to the species *L. ivanovii*, though the isolation of this species was not frequent. Among 341 intestinal fecal samples of rodents captured from five different regions of China, seven were positive for *L. ivanovii*. All of these came from animals captured in Tibet; five at the junction of farm area and woodland and two in a grassland. Three isolates derived from *A. peninsulae*, two from *Cricetulus kamensis* and two from *N. confucianus* [39]. Cao et al. [25] isolated 26 *L. ivanovii* strains from 702 fecal samples of 25 different species of wild rodents from six provinces of China. The isolates were assigned to 5 STs with ST6 being the dominant type. The prevalence of *L. ivanovii* was higher in some regions, and the genetic diversity was relatively low since most isolates belonged to one lineage. 

In an investigation carried out in Turkey, *L. ivanovii* was isolated from the abomasum content of an aborted fetus from a farm with history of silage feeding, among 538 analyzed specimens comprising 229 milk samples, 263 vaginal swabs and 46 abomasum contents of aborted sheep fetuses. In another sample of abomasum content from an aborted fetus *L. ivanovii* was identified by direct application of genus-specific PCR and subsequent 16S rRNA gene sequencing [40].

Abuhatab et al. [41] reported that *L. ivanovii* was the most prevalent species isolated from cloacal swabs of avian species in a study carried out in Egypt. This species was found in 32% samples from broilers, layers, pigeons, ducks and turkeys and was isolated from all these animals. Moreover, it was isolated from two of eight chicken carcasses, one of four chicken luncheons, one of three frozen chicken breast fillets, 2 of 9 eggshells and one of two fecal specimens from poultry farm workers. Molecular identification tests were carried out only for the *L. monocytogenes* isolates. This study suggested to further investigate the occurrence of *L. ivanovii* in avian species.

In an investigation carried out in all the operational units of an Ethiopian university dairy farm, *L. ivanovii*, identified on the basis of biochemical tests, was not isolated from feed (silage) but from the milk harvesting cylinder, pooled milk at collection and supply and milk measuring equipment in one or two out of 10 samples. It could not be isolated from cow barn and milking parlor floors, drinking and cleaning water and teat drying towels. Therefore, it is possible that the milk harvesting cylinder was contaminated by a persistent *L. ivanovii* strain that was released into the milk [42]. 

In Latvia, *L. ivanovii* was isolated from 2 among 136 water samples from river and farm water and 3 of 111 animal feces samples in cattle farms [43].

Deer and wild boars were indicated as natural reservoirs of *L. ivanovii* in a study in which the subsp. *londoniensis* was detected in 4 among 23 tonsil samples [44].

In an analysis of *Listeria* sensu stricto species distribution in publicly available metagenomic datasets from the large MG-RAST database 11,907, 16S rRNA sequence high-quality datasets were examined [45]. *L. ivanovii* specific sequences were detected in soil, human and animal hosts, sludge and sediments. It was the second abundant species in humans, particularly in datasets from gut and skin, cow and goat associated environments. This finding indicates that the culture-dependent examination allows the isolation of *L. ivanovii* only from a subset of samples in which it is present. In addition, only *L. ivanovii* was detected in 16S rRNA datasets from goats, confirming the association of *L. ivanovii* with small ruminants [46]. 

## 6. *L. ivanovii* Infection Cases in Humans

Guillet et al. [11], while reporting a case of *L. ivanovii* subsp. *ivanovii* serovar 5 gastroenteritis and bacteremia in a kidney-transplanted patient of 55 years of age in immunosuppressive regimen, reviewed the previous literature regarding infections caused by this bacterial species before 2010. Since 1985, they found three well-documented cases of *L. ivanovii*–human infection, one febrile diarrhea and two bacteremia cases. The infections were associated with Acquired Immune Deficiency Syndrome (AIDS), metastatic carcinoma or substance abuse. Two patients were more than 60 years old. Therefore, as for *L. monocytogenes*, human *L. ivanovii* infection is associated with immunodeficiency, underlying debilitating conditions, or advanced age. In other instances, *L. ivanovii* was isolated from human samples, in two cases fetoplacental tissue and lochia and in one case a mesenteric lymph node. The pathologic changes caused by *L. ivanovii* in humans appeared similar to those in ruminants, i.e., fetoplacental infections and septicemia often accompanied by enteritis. Lack of central nervous system involvement could be a general characteristic of *L. ivanovii* infection regardless of host species.

Beye et al. [10] reported the first case of *L. ivanovii* human vascular infection in a 78-year-old man in 2015 who underwent to two cardiac and one aortic surgical intervention a few years earlier from which strain *L. ivanovii* G770 was isolated.

A recent case report regarded the isolation of *L. ivanovii* subsp. *ivanovii* CC883 in a case of chronic lymphadenitis without sign of malignancy in which the right iliac lymphnode was affected in an eleven-year-old boy experiencing fever, anorexia and abdominal pain. The case was resolved after surgical lymphnode removal and treatment with amoxicillin. Other ten cases reported by the same study and eight cases of listerial lymphadenitis documented by the literature were instead caused by *L. monocytogenes* [12].

## 7. *L. ivanovii* Infection Cases in Animals

*L. ivanovii* is considered pathogenic mainly for ruminants [47], but the real occurrence of infections caused by this bacterium in animals is not well documented in literature. However, some recent outbreaks were reported. One of these regard visceral *L. ivanovii* infection in seven weaned lambs from five farms, examined postmortem at Veterinary Investigation Centres of the Animal and Plant Health Agency, UK, between September 2018 and January 2019. All animals were affected by a concurrent parasitic gastroenteritis that was therefore suggested to be a debilitating condition exposing to *L. ivanovii* infection [48]. 

It was also reported that *L. ivanovii* caused abortion in ten Santa Inés ewes over a period of one month in a flock of 390 heads in Santa Fe, Argentina. Aborted fetuses were full-term and covered by the fetal membranes. A fetus aborted at 130 days of gestation exhibited necrotizing hepatitis, suppurative bronchopneumonia, diffuse meningitis and occasional foci of gliosis in the brainstem and spinal cord. Bacterial colonies were present in the liver, lungs and meninges lesions. *L. ivanovii* was isolated from the placenta, brain, liver, lung and abomasal content. It could not be isolated from the maize silage fed to the animals but there were no more abortions after that the administration of this feed to pregnant ewes ceased and the feeding equipment was disinfected [49]. The latter report indicates that outbreaks of *L. ivanovii* infections could arise when animals are exposed to high loads of the pathogen, as in the case of being fed with contaminated silage.

## 8. Occurrence of *L. ivanovii* in Food

To evaluate the distribution of *L. ivanovii* in food products, we performed a bibliographic search for all the reports regarding the distribution of *L. monocytogenes*/*Listeria* spp. in different food categories. These were screened to select those reporting the isolation of the species *L. ivanovii*. Reports retrieved, with type and number of positive samples and percentage of positive samples on the total number of samples analyzed (positivity rate) are summarized in Table 1, with indication of the countries in which the investigations were carried out and respective references. Further details on single studies, such as the number of samples analyzed, the analytical procedure adopted, and the specific food products examined, are reported in Table S1. 

We can point out that *L. ivanovii* was found to be present in numerous food categories of both animal and plant origin, indicating a distribution of this species in different environments. Most reports regard African countries, mainly Egypt, followed by Nigeria and Ethiopia, where high positivity rates were sometimes observed. This parameter ranged between a minimum of 0.1% and a maximum of 35% for a study on raw cow milk in Jordan.

Reports with the higher *L. ivanovii* prevalence values regarded countries where small ruminant farming is widely practiced. However, no reports were found for countries such as New Zealand and Australia where small ruminant raising is also common. This can be an indication of under-reporting of the occurrence of *L. ivanovii*. Indeed, this bacterium is probably still present in those countries where the first representative strains of the species were isolated [153,154]. 

The higher positivity rates reported in some studies cannot be easily interpreted and should be corroborated by investigations on optimal growth conditions of *L. ivanovii* and characterization of isolates on this respect. 

**Figure 1:**
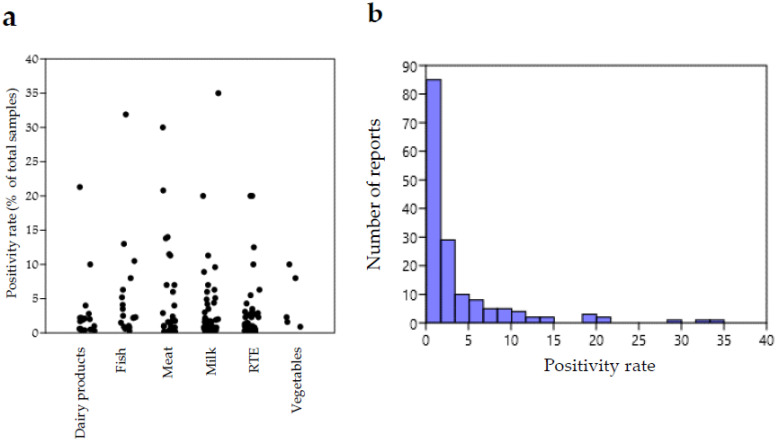
(**a**) Jitter plot representation of the distribution of reports summarized in Table 1 according to positivity rate (percentages of positive samples on total number of analyzed samples) per food category. Each circle corresponds to a report. (**b**) Histogram showing the number of reports falling in different intervals of *L. ivanovii* positivity rate for all food categories (B). Plots were obtained with the Past Statistical software 4.03 downloadable at https://past.en.lo4d.com/windows, accessed on 15 July 2022 [155].

Figure 1 shows graphical representations of the distribution of *L. ivanovii* occurrence reports according to positivity rate. Figure 1a shows a plot of the distribution of *L. ivanovii* reports summarized in Table 1 per positivity rate and food category, whereas Figure 1b shows the distribution of all the reports according to the positivity rate. 

It can be observed that the distribution of positivity rates was not very variable for different food categories, except for a few investigations reporting exceptionally high values. This suggests that the risk of contamination by *L. ivanovii* does not differ remarkably among food types.

In addition to the reports summarized in Table 1, further information on the occurrence of *L. ivanovii* in food is provided by a recent systematic review reporting that *L. ivanovii* was the predominant *Listeria* species isolated from foods in Ethiopia, including cheese, raw milk, raw beef, ice cream and eggs [156].

Finally, in a study evaluating the microbiological quality of dry pet snacks, the presence of *L. ivanovii* was reported in 1 among 120 samples analyzed, while the other 119 samples appeared in good sanitary condition [157].

## 9. Methods of Isolation and Identification of *L. ivanovii*

### 9.1. Isolation Methods

In most of the studies reviewed here, *L. ivanovii* was isolated by using culture dependent standardized procedures that are considered to allow the recovery of all *Listeria* species (Table S1). However, a recent investigation highlighted that *L. ivanovii* has a lower growth capacity than other *Listeria* species in some enrichment media currently used [1]. The study aimed to assessing the inclusivity of the selective broths specified by the U.S. Food and Drug Administration (FDA) [158], the International Organization for Standardization (ISO) [159], and the U.S. Department of Agriculture, Food Safety and Inspection Service (USDA) [160] methods for strains representing 6 *Listeria* sensu stricto and 13 *Listeria* sensu lato species and variations in colony morphology on the selective and differential agar media. The study highlighted that with the USDA and ISO broth enrichment procedures, several *Listeria* sensu lato showed a significantly higher growth than *L. seeligeri* and *L. ivanovii*, suggesting that these two *Listeria* sensu stricto species could be outgrown by *Listeria* sensu lato species when analyzing real samples. In 24 h of selective enrichment, only buffered *Listeria* enrichment broth (BLEB) supported the growth of all 19 species to more than 4 log CFU/mL, whereas some species grew between 1 and 4 log CFU/mL) in Demi Fraser, Fraser, 3-(N-morpholino) propanesulfonic acid (MOPS) BLEB, and University of Vermont medium (UVM). *L. ivanovii* had a limited growth in Fraser broth and growth tests in co-culture showed that *L. ivanovii* had a significantly lower growth than all the other *Listeria* species. This study suggests that this species may be outgrown by another species during selective enrichment, except in BLEB, after 48 h. Data suggested that *L. ivanovii* detection with the ISO method may be challenging because the secondary enrichment Fraser medium supported only limited growth of this species, even though growth was high in the primary enrichment culture. A previous study highlighted that *L. ivanovii* did not grow in Fraser broth after 24 h [161] and in a recent investigation it was found that three *L. ivanovii* isolates from ovine bulk tank milk did not form colonies on agar *Listeria* Ottavani & Agosti (ALOA) after the first enrichment in half-Fraser and formed a few colonies after the second enrichment step in Fraser broth on this medium. A better growth was obtained on modified Oxford agar (MOX) after both enrichment steps [127].

On the other hand, Carlin et al. [1] reported that *L. ivanovii* originated typical colonies on ALOA, i.e., 1 to 3 mm in diameter, round, regular, and blue-green with opaque halos formed for the phosphatidylinositol-specific phospholipase C (PI-PLC) activity and on *L. monocytogenes* chromogenic plating medium (LMCPM), namely convex blue-green colonies indicative of PI-PLC activity 1 to 3 mm in diameter, but formed atypical colonies on MOX, appearing to be partially inhibited. Therefore, this medium might not be suitable for *L. ivanovii* isolation.

### 9.2. Molecular Identification and Detection

A species-specific conventional PCR test for *L. ivanovii*, targeted on a cloned fragment from this species, is available and allows identification based on the amplification of a 463 bp band [162]. In addition, different multiplex PCR tests are available that simultaneously detect *L. ivanovii* and *L. monocytogenes*. For instance, the species *L. ivanovii* can be identified by a multiplex PCR able to amplify multiple internalin genes of *L. monocytogenes inl*A, *inl*C and *inl*J, because it gives a positive reaction only for *inl*C [163]. 

A different multiplex PCR assay was designed for the identification of pathogenic *Listeria*, with primers targeting the genes specific for *Listeria* genus (LMOSLCC2755_0944), *L*. *monocytogenes* (LMOSLCC2755_0090), and *L. ivanovii* queuosine precursor ECF transporter S component *que*T_1 and was used to analyze samples of the mushroom *Flammulina velutipes* following a 4–12 h enrichment [164].

In another method, *L. ivanovii* specific primers were designed on the gene *iact*A and used in a duplex reaction for the simultaneous detection of *L. ivanovii* and *L*. *monocytogenes*. The test was applied to detection in lettuce following immunomagnetic separation with optimized amounts of streptavidin and biotinylated anti-*Listeria* monoclonal antibodies coated magnetic nanobeads [165]. The time of analysis was less than 7 h and the limit of detection was 1.0 CFU/mL in pure culture and 10 CFU/g in lettuce.

Xiao et al. [166] used surface-modified polyethyleneimine-coated positively charged magnetic nanoparticles (PEI-MNPs) for rapid enrichment of pathogenic *Listeria* spp. through electrostatic interactions. The enrichment process takes only 10 min with more than 70% capture efficiency at wide ranges of pH and ionic strength. In the method development, a multiplex PCR comprising the primers designed by Mao et al. [165] for *L. ivanovii*, primers specific for *L*. *monocytogenes* and universal primers for bacteria was applied. The PEI-MNPs-mPCR combination did not require pre-concentration and permitted to detect 10 CFU/mL of both *Listeria* species in lettuce suspension. 

Real Time PCR was applied by Rodríguez-Lázaro et al. [167] for detection/quantification of *L. ivanovii* using the *smc*L gene as target. The method allowed to detect 50 CFU of the bacterium in 25 mL of raw milk, 43 CFU in 1 mL of blood and 50 CFU in 1 mL of amniotic fluid.

A loop-mediated isothermal amplification (LAMP) assay for rapid and sensitive detection of *L. ivanovii* was also designed on the *smc*L gene and allowed to detect 16 CFU per reaction of *L. ivanovii* in pure cultures and simulated human stool. This LAMP assay allowed to detect 8 CFU/0.5 g of *L. ivanovii* spiked in human stool samples after 6 h enrichment, so that it could be conveniently used for the detection of *L. ivanovii* in field, medical and veterinary laboratories [168].

As a rapid identification technique, matrix-assisted laser desorption/ionization time-of-flight mass spectrometry (MALDI-TOF MS) proved able to discriminate *L. ivanovii* and the other species of the *Listeria* sensu stricto clade [169].

## 10. Conclusions

In this review, updated knowledge was gathered on pathogenicity and occurrence in the environment and in food of *L. ivanovii*. The evidence collected still seems to indicate that this bacterium presents the risk to cause disease in immune-compromised hosts. Virulence was reported to be variable among strains based on the presence/absence and variability of genomic traits. Moreover, the distribution in food appeared to be wide and maybe underestimated for the limited suitability of some culture media used in standard analytical procedures to allow its growth. In addition, focused investigations on this species were very few, so its full pathogenic potential is still undiscovered. Therefore, the optimization of the analytical methods for the isolation of *L. ivanovii* should be undertaken as well as the isolation and genome sequencing and analysis for a high number of strains.

## Figures and Tables

**Table 1:** Food products in which the presence of *L. ivanovii* was reported, number of positive samples, positivity rate (percentage of positive samples on the total number of samples analyzed in each study), country of isolation and reference.

Food Matrix	Number of Samples Positive for *L. ivanovii*, Positivity Rate (%) on All Analyzed Samples for Each Report, Country and Reference
Dairy products	
Butter	1 (0.4) Egypt [50], 1 (0.3) Turkey [51]
Cheese	3 (0.6) Portugal [52], 3 (1.9) Turkey [53], 32 (2.2) Colombia [54], 3 (2.1) Turkey [55], 2 (2.8) Italy [56], 2 (0.2) Ethiopia [57], 1 (1.7) Turkey [58], 3 (10) Venezuela [59], 1 (0.4) Turkey [60], 2 (2.7) Turkey [61], 14 (4) Jordan [62], 1 (0.4) Iraq [63], 1 (0.4) Egypt [50], 6 (2.2) Turkey [64], 2 (0.6) Turkey [51], 6 (0.5) Turkey [65], 5 (4.0) Turkey [66], 4 (2.0) Turkey [67], 2 (1.0) Libya [68], 48 (21.3) Egypt [69]
Fish	
Conserved fish and seafood	2 (5.2) India [70], 2 (0.6) Spain [71], 30 (31.9) Malaysia [72], 15 (13) Nigeria [73]
Fresh or frozen fish and seafood,	2 (0.3) Japan [74], 4 (10.5) India [70], 3 (2.2) Costa Rica [75], 5 (2.3) Italy [76], 1 (1) Turkey [77], 7 (3.5) Iran [78], 3 (6.3) Greece [79], 3 (1) Iran [80], 2 (0.9) Egypt [81], 8 (8) Egypt [82], 8 (2.2) Iran [83], 21 (4.1) Jordan [84], 2 (0.8) USA [85], 3 (1.5) Libya [68]
Meat	
Conserved chicken	2 (7) India [70], 1 (1) Egypt [86], 25 (20.8) Jordan [87]
Conserved pork	4 (14) India [70], 2 (0.2) Bulgaria [88], 1 (1.0) Turkey [89]
Conserved meat (animal species not detailed)	1 (0.2) Ireland [26]
Raw/frozen beef	14 (13.8) Malaysia [72], 1 (0.3) Ethiopia [90], 2 (0.2) Bulgaria [89], 2 (0.2) Ethiopia [57], 1 (0.25) Ethiopia [91], 4 (1.7) Nigeria [92], 3 (1.6) Egypt [93], 1 (0.2) Brazil [94], 6 (1.8) Nigeria [95], 2 (0.8) Egypt [96], 5 (4.0) Turkey [97], 7 (2.9) Nigeria [98], 1 (0.5) Libya [68], 12 (11.5) Nigeria [99], 4 (0.9) Ethiopia [100], 4 (0.9) Nigeria [101]
Raw buffalo meat	1 (1) Egypt [102]
Raw/frozen chicken	2 (7) India [70], 6 (6) Egypt [86], 48 (30.0) Jordan [87], 1 (1.0) Spain [103], 10 (2.4) Iran [104], 2 (1.7) Nigeria [105], 9 (11.3) Egypt [106], 5 (2.0) Nigeria [98], 3 (0.6) Ethiopia [100], 3 (0.6) Nigeria [101], 4 (27) Egypt [41]
Raw/frozen lamb	1 (1.4) Brazil [107],
Raw goat meat	10 (9.6) Nigeria [99]
Raw/frozen meat (animal species not detailed)	9 (8.9) Malaysia [72], 10 (1.7) Italy [108], 19 (7.0) Jordan [109], 2 (0.5) Ethiopia [110], 17 (5.1) Nigeria [95], 10 (2.2) Ethiopia [100], 1 (0.9) India [111]
Raw/frozen pork	2 (1.8) Spain [112], 1 (0.1) Bulgaria [88], 17 (11.3) Italy [113]
Raw rabbit	1 (2.0) Spain [114]
Milk	
Raw buffalo milk	1 (1.6) Egypt [115], 2 (0.8) Egypt [116]
Raw cow milk	2 (0.4) Portugal [51], 82 (6.3) Mexico [117], 7 (35.0) Jordan [118], 8 (4.2) Nigeria [119], 7 (0.9) Syria [120], 1 (0.5) Egypt [93], 1 (0.4) Iran [121], 1 (0.5) Turkey [122], 1 (1.6) Egypt [115], 12 (6) Egypt [123], 2 (0.8) Egypt [116], 3 (4.4) Turkey [124]
Raw ewe milk	2 (0.2) Spain [125], 5 (0.9) Spain [126], 5 (0.6) Syria [120], 1 (0.4) Iran [121], 1 (1.6) Egypt [115], 1 (0.4) Egypt [116], 3 (0.9) Greece [127]
Raw goat milk	3 (0.2) Spain [128], 4 (20) Egypt [129]
Raw zebu milk	2 (1.9) Tanzania [130]
Raw milk (animal species not detailed)	1 (0.6) Turkey [53], 1 (0.4) Italy [56], 1 (0.9) India [127], 1 (0.4) Turkey [60], 2 (0.8) Egypt [50], 27 (4.9) Nigeria [131], 5 (1.6) Turkey [51], 1 (0.9) Turkey [66], 3 (1.5) Turkey [67], 7 (3.5) Libya [68], 2 (1.7) Sudan [132], 6 (1.2) Ethiopia [133]
Raw buffalo milk	1 (1.6) Egypt [115], 2 (0.8) Egypt [116]
Ready to eat (RTE) ^1^ food products	
Meat (animal species not detailed)	1 (0.2) Portugal [52], 3 (0.7) Iran [104], 1 (0.4) Egypt [134], 12 (3.5) Nigeria [135], 19 (6.3) Nigeria [136], 54 (20) Jordan [109], 1 (0.4) Egypt [50], 12 (10) Egypt [137], 2 (1.0) Libya [68]
Beef	6 (2.3) South Africa [138], 4 (1.4) Turkey [139], 8 (2.7) Egypt [140], 1 (0.8) Egypt [141]
Cabbages	1 (1.0) Croatia [142], 9 (2.6) Nigeria [135],
Cheese	1 (0.3) Turkey [139],
Chicken	11 (4.3) South Africa [138], 52 (20.8) Sudan [143],
Lettuce	3 (2.9) Spain [112], 1 (0.5) Italy [144], 3 (3.0) Croatia [142], 10 (2.9) Nigeria [135]
Potatoes	6 (2.3) South Africa [138]
Rice	6 (2.3) South Africa [138]
Bean sprouts	1 (0.1) Ireland [145],
Turkey	1 (0.3) Egypt [140]
Vegetables	8 (3.1) South Africa [138]
Not detailed	3 (0.8) Thailand [146], 20 (20.0) Jordan [118], 3 (1.3) Algeria [147], 4 (1) Taiwan [148], 1 (1.0) Croatia [142], 14 (5.5) South Africa [138], 6 (1.5) Nigeria [149], 3 (12.5) Italy [150]
Vegetables	
Coriander	32 (1.6) Venezuela [151]
Leafy vegetables	1 (10) Nigeria [152]
Lettuce	1 (0.9) Spain [112]
Tomato	16 (8.0) Venezuela [151]
Not detailed	17 (2.3) Nigeria [95]
Other	
Liquid whole egg	1 (0.25) Ethiopia [91]

^1^ listed according to the main component.

## Supplementary Materials

The following supporting information can be downloaded at: https://www.mdpi.com/article/10.3390/microorganisms10081679/s1, Table S1: Reports of *L. ivanovii* isolation from food products in chronological order with type of food, total number of samples tested in the same study, country, analytical methods used and reference.

## References

[1] Carlin C.R., Roof S., Wiedmann M. (2022). Assessment of Reference Method Selective Broth and Plating Media with 19 *Listeria* Species Highlights the Importance of Including Diverse Species in *Listeria* Method Evaluations. J. Food Prot..

[2] Orsi R.H., Wiedmann M. (2016). Characteristics and distribution of *Listeria* spp., including *Listeria* species newly described since 2009. Appl. Microbiol. Biotechnol..

[3] Vázquez-Boland J.A., Kuhn M., Berche P., Chakraborty T., Domínguez-Bernal G., Goebel W., González-Zorn B., Wehland J., Kreft J. (2001). *Listeria* pathogenesis and molecular virulence determinants. Clin. Microbiol. Rev..

[4] Schmid M.W., Ng E.Y., Lampidis R., Emmerth M., Walcher M., Kreft J., Goebel W., Wagner M., Schleifer K.H. (2005). Evolutionary history of the genus *Listeria* and its virulence genes. Syst. Appl. Microbiol..

[5] Ivanov I., Woodbine M. (1975). Establishment of non-motile strains of *Listeria monocytogenes* type 5. Problems of Listeriosis.

[6] Seeliger H.P.R., Welshimer H.J., Buchanan R.E., Gibbons N.E. (1974). Genus *Listeria*. Bergey’s Manual of Determinative Bacteriology.

[7] Seeliger H.P.R., Rocourt J., Schrettenbrunner A., Grimont P.A.D., Jones D. (1984). Notes: *Listeria ivanovii* sp. nov. Int. J. Syst. Evolut. Microbiol..

[8] Boerlin P., Rocourt J., Grimont F., Grimont P.A.D., Jaquet C., Piffaretti J.C. (1992). *Listeria ivanovii* subsp. *londoniensis* subsp. nov. Int. J. Syst. Evolut. Microbiol..

[9] Hupfeld M., Trasanidou D., Ramazzini L., Klumpp J., Loessner M.J., Kilcher S.A. (2018). functional type II-A CRISPR-Cas system from *Listeria* enables efficient genome editing of large non-integrating bacteriophage. Nucleic Acids Res..

[10] Beye M., Gouriet F., Michelle C., Casalta J.P., Habib G., Raoult D., Fournier P.E. (2016). Genome analysis of *Listeria ivanovii* strain G770 that caused a deadly aortic prosthesis infection. New Microbes New Infect..

[11] Guillet C., Join-Lambert O., Le Monnier A., Leclercq A., Mechai F., Mamzer-Bruneel M.F., Bielecka M.K., Scortti M., Disson O., Berche P. (2010). Human listeriosis caused by *Listeria ivanovii*. Emerg. Infect. Dis..

[12] Blot M., Disson O., Leclercq A., Moura A., Bracq-Dieye H., Thouvenot P., Valès G., Burroni B., Lupo A., Lecuit M. (2022). *Listeria*-Associated Lymphadenitis: A Series of 11 Consecutive Cases and Review of the Literature. Open Forum Infectious Diseases.

[13] Gan L., Mao P., Jiang H., Zhang L., Liu D., Cao X., Wang Y., Wang Y., Sun H., Huang Y. (2020). Two prevalent *Listeria ivanovii* subsp. *ivanovii* clonal strains with different virulence exist in wild rodents and pikas of China. Front. Vet. Sci..

[14] Domínguez-Bernal G., Müller-Altrock S., González-Zorn B., Scortti M., Herrmann P., Monzó G.H., Lacharme L., Kreft J., Vázquez-Boland A.J. (2006). A spontaneous genomic deletion in *Listeria ivanovii* identifies LIPI-2, a species-specific pathogenicity island encoding sphingomyelinase and numerous internalins. Mol. Microbiol..

[15] Gedde M.M., Higgins D.E., Tilney L.G., Portnoy D.A. (2000). Role of listeriolysin O in cell-to-cell spread of *Listeria monocytogenes*. Infect. Immun..

[16] Buchrieser C., Rusniok C., Garrido P., Hain T., Scortti M., Lampidis R., Kärst U., Chakraborty T., Cossart P., Kreft J. (2011). Complete genome sequence of the animal pathogen *Listeria ivanovii*, which provides insights into host specificities and evolution of the genus *Listeria*. J. Bacteriol..

[17] Disson O., Lecuit M. (2013). In vitro and in vivo models to study human listeriosis: Mind the gap. Microb. Infect..

[18] Van Ngo H., Bhalla M., Chen D.Y., Ireton K. (2017). A role for host cell exocytosis in InlB-mediated internalization of *Listeria monocytogenes*. Cell. Microbiol..

[19] Engelbrecht F., Domínguez-Bernal G., Hess J., Dickneite C., Greiffenberg L., Lampidis R., Raffelsbauer D., Daniels J.J., Kreft J., Kaufmann S.H. (1998). A novel PrfA-regulated chromosomal locus, which is specific for *Listeria ivanovii*, encodes two small, secreted internalins and contributes to virulence in mice. Mol. Microbiol..

[20] González-Zorn B., Domínguez-Bernal G., Suárez M., Ripio M.T., Vega Y., Novella S., Rodríguez A., Chico I., Tierrez A., Vázquez-Boland J.A. (2000). SmcL, a novel membrane-damaging virulence factor in *Listeria*. Int. J. Med. Microbiol..

[21] Gouin E., Dehoux P., Mengaud J., Kocks C., Cossart P. (1995). iactA of *Listeria ivanovii*, although distantly related to *Listeria monocytogenes* actA, restores actin tail formation in an *L. monocytogenes* actA mutant. Infect. Immun..

[22] Karunasagar I., Krohne G., Goebel W. (1993). *Listeria ivanovii* is capable of cell-to-cell spread involving actin polymerization. Infect. Immun..

[23] Hain T., Chatterjee S.S., Ghai R., Kuenne C.T., Billion A., Steinweg C., Domann E., Kärst U., Jänsch L., Wehland J. (2007). Pathogenomics of *Listeria* spp. Int. J. Med. Microbiol..

[24] Kuenne C., Billion A., Mraheil M.A., Strittmatter A., Daniel R., Goesmann A., Barbuddhe S., Hain T., Chakraborty T. (2013). Reassessment of the *Listeria monocytogenes* pan-genome reveals dynamic integration hotspots and mobile genetic elements as major components of the accessory genome. BMC Genom..

[25] Cao X., Wang Y., Wang Y., Li H., Luo L., Wang P., Zhang L., Li H., Liu J., Lu L. (2019). Prevalence and Characteristics of *Listeria ivanovii* Strains in Wild Rodents in China. Vector Borne Zoonotic Dis..

[26] Alvarez-Ordóñez A., Leong D., Morgan C.A., Hill C., Gahan C.G., Jordan K. (2015). Occurrence, persistence, and virulence potential of *Listeria ivanovii* in foods and food processing environments in the Republic of Ireland. BioMed. Res. Int..

[27] Ammendolia M.G., Superti F., Bertuccini L., Chiarini F., Conte M.P., Cipriani D., Seganti L., Longhi C. (2007). Invasive pathway of *Listeria ivanovii* in human amnion-derived WISH cells. Int. J. Immunopathol. Pharmacol..

[28] Rocha C.E., Mol J.P.S., Garcia L.N.N., Costa L.F., Santos R.L., Paixao T.A. (2017). Comparative experimental infection of *Listeria monocytogenes* and *Listeria ivanovii* in bovine trophoblasts. PLoS ONE.

[29] Zhou M., Jiang M., Ren C., Liu S., Pu Q., Goldfine H., Shen H., Wang C. (2016). *Listeria ivanovii* Infection in Mice: Restricted to the Liver and Lung with Limited Replication in the Spleen. Front. Microbiol..

[30] Borovuk I., Zazharska N. (2022). Evaluation of broiler meat in experimental listeriosis. J. Adv. Vet. Anim. Res..

[31] Nyenje M.E., Green E., Ndip R.N. (2012). Biofilm formation and adherence characteristics of *Listeria ivanovii* strains isolated from ready-to-eat foods in Alice, South Africa. Scient. World J..

[32] Vázquez-Villanueva J., Orgaz B., Ortiz S., López V., Martínez-Suárez J.V., Sanjose C. (2010). Predominance and persistence of a single clone of *Listeria ivanovii* in a Manchego cheese factory over 6 months. Zoonoses Public Health.

[33] Chmielowska C., Korsa D., Szmulkowska B., Krop A., Lipka K., Krupińska M., Bartosik D. (2020). Genetic Carriers and Genomic Distribution of cadA6-A Novel Variant of a Cadmium Resistance Determinant Identified in *Listeria* spp. Int. J. Mol. Sci..

[34] Müller A., Rychli K., Zaiser A., Wieser C., Wagner M., Schmitz-Esser S. (2014). The *Listeria monocytogenes* transposon Tn6188 provides increased tolerance to various quaternary ammonium compounds and ethidium bromide. FEMS Microbiol. Lett..

[35] Bolten S., Harrand A.S., Skeens J., Wiedmann M. (2022). Nonsynonymous Mutations in *fepR* Are Associated with Adaptation of *Listeria monocytogenes* and Other *Listeria* spp. to Low Concentrations of Benzalkonium Chloride but Do Not Increase Survival of *L. monocytogenes* and Other *Listeria* spp. after Exposure to Benzalkonium Chloride Concentrations Recommended for Use in Food Processing Environments. Appl. Environ. Microbiol..

[36] Longhi C., Ammendolia M.G., Conte M., Seganti L., Iosi F., Superti F. (2014). *Listeria ivanovii* ATCC 19119 strain behaviour is modulated by iron and acid stress. Food Microbiol..

[37] Rawool D.B., Malik S.V.S., Shakuntala I., Sahare A.M., Barbuddhe S.B. (2007). Detection of multiple virulence-associated genes in *Listeria monocytogenes* isolated from bovine mastitis cases. Int. J. Food Microbiol..

[38] Elezebeth G., Malik S.V.S., Chaudhari S.P., Barbuddhe S.B. (2007). The occurrence of *Listeria* species and antibodies against listeriolysin-O in naturally infected goats. Small Rumin. Res..

[39] Wang Y., Lu L., Lan R., Salazar J.K., Liu J., Xu J., Ye C. (2017). Isolation and characterization of *Listeria* species from rodents in natural environments in China. Emerg. Microbes Infect..

[40] Akca D., Buyuk F., Celik E., Saglam A.G., Otlu S., Dag S., Celebi O., Coskun M.R., Buyuk E., Karakurt E. (2022). Phylogenetic positioning of *Listeria ivanovii* identified in aborted sheep in Kars Region (Turkey). Thai J. Vet. Med..

[41] Abuhatab E., Naguib D., Abdou A., Gwida M., Elgohary A. (2022). Genetic Characterization and Antibiogram Profiles of *Listeria* species Isolated from Poultry and Poultry Handlers. J. Adv. Vet. Res..

[42] Ahimeda H.M., Hikoa A., Abdellaha A., Muktarb Y.D., Gutema F.D. (2022). Isolation and multidrug drug resistance profile of *Listeria* species in selected Dairy Farm’s Operational stages in Oromia Regional State, Ethiopia. Sci. Afr..

[43] Terentjeva M., Šteingolde Z., Meistere I., Elferts D., Avsejenko J., Streikiša M., Gradovska S., Alksne L., Ķibilds J., Bērziņš A. (2021). Prevalence, Genetic Diversity and Factors Associated with Distribution of *Listeria monocytogenes* and Other *Listeria* spp. in Cattle Farms in Latvia. Pathogens..

[44] Palacios-Gorba C., Moura A., Leclercq A., Gómez-Martín A., Gomis J., Jiménez-Trigos E., Mocé M.L., Lecuit M., Quereda J.J. (2021). *Listeria* spp. Isolated from Tonsils of Wild Deer and Boars: Genomic Characterization. Appl. Environ. Microbiol..

[45] Meshref L., Pichon M., Burucoa C., Nusser S.H.A., Moura A., Garcia-Garcera M., Lecuit M. (2021). *Listeria monocytogenes* faecal carriage is common and depends on the gut microbiota. Nat. Commun..

[46] Ramage C.P., Low J.C., McLauchlin J., Donachie W. (2006). Characterisation of *Listeria ivanovii* isolates from the UK using pulsed-field gel electrophoresis. FEMS Microbiol. Lett..

[47] Low J.C., Donachie W. (1997). A Review of *Listeria monocytogenes* and Listeriosis. Vet. J..

[48] Dunnett E., Florea L., Thurston L., Floyd T., Collins R., Otter A. (2020). Deaths of weaned lambs with visceral *Listeria ivanovii* infections. Vet. Rec. Case Rep..

[49] Della Rosa P., Colque Caro L.A., Cantón G.J., Morrell E.L., Hecker Y.P., Paolicchi F.A., Fiorentino M.A. Aborto ovino asociado a *Listeria ivanovii*. Proceedings of the XV Congreso Argentino de Microbiología.

[50] Meshref A., Zeinhom M., Abdel-Atty N.S. (2015). Occurrence and distribution of *Listeria* species in some Egyptian foods. Alex. J. Vet. Sci..

[51] Aksoy A., Sezer Ç., Vatansever L., Gulbaz G. (2018). Presence and antibiotic resistance of *Listeria monocytogenes* in raw milk and dairy products. Kafkas Üniversitesi Veteriner Fakültesi Dergisi.

[52] Guerra M.M., McLauchlin J., Bernardo F.A. (2001). *Listeria* in ready-to-eat and unprocessed foods produced in Portugal. Food Microbiol..

[53] Aygun O., Pehlivanlar S. (2006). *Listeria* spp. in the raw milk and dairy products in Antakya, Turkey. Food Control.

[54] Gallegos J.M., Vanegas M.C., Albarracín Y., Máttar S., Poutou R.A., Carrascal A.K. (2008). Frequency of isolation of *Listeria* species in different retail foods in Colombia. Anim. Prod. Res. Adv..

[55] Arslan S., Özdemir F. (2008). Prevalence and antimicrobial resistance of *Listeria* spp. in homemade white cheese. Food Control.

[56] Latorre L., Fraccalvieri R., Parisi S., Santagada G., Normanno G. (2008). Studio sulla contaminazione da *Listeria* spp. e *Listeria monocytogenes* in latte e prodotti lattiero-caseari ovi-caprini. Ind. Aliment..

[57] Mengesha D., Zewde B.M., Toquin M.T., Kleer J., Hildebrandt G., Gebreyes W.A. (2009). Lebensmittelhygiene-Vorkommen und Verteilung von *Listeria monocytogenes* und anderen *Listeria* spp. in verzehrsfertigen Lebensmitteln und rohem Fleisch. Berliner und Munchener Tierarztliche Wochenschrift.

[58] Büyükyörük S., Göksoy E.Ö. (2011). Aydın ilinde satışa sunulan köy peynirlerinde *Listeria* varlığının araştırılması. Uludağ Üniversitesi Vet. Fakültesi Derg..

[59] Ramírez Mérida L.G., Morón de Salim A., Alfieri Graterol A.Y., Gamboa O. (2010). Detección de *Listeria monocytogenes* en queso blanco criollo, mediante la reacción en cadena de la polimerasa (PCR). Archivos Latinoamericanos de Nutrición.

[60] Sağun E., Sancak Y.C., İşleyici Ö., Ekici K. (2001). The presence and prevalence of *Listeria* species in milk and herby cheese in and around Van. Turk. J. Vet. Anim. Sci..

[61] Cokal Y., Dagdelen A., Cenet O., Gunsen U. (2012). Presence of *L. monocytogenes* and some bacterial pathogens in two Turkish traditional foods, Mihalic cheese and Hosmerim dessert. Food Control.

[62] Osaili T.M., Al-Nabulsi A.A., Taha M.H., Al-Holy M.A., Alaboudi A.R., Al-Rousan W.M., Shaker R.R. (2012). Occurrence and antimicrobial susceptibility of *Listeria monocytogenes* isolated from brined white cheese in Jordan. J. Food Sci..

[63] Alzubaidy Z.M., Kakey S.I., Ali J.F. (2013). Isolation and identification of *Listeria moncytogenes* by PCR from some food sources in Erbil city. Euphrates J. Agric. Sci..

[64] Kaptan B. (2016). Prevalence of *Listeria* spp. and *L. monocytogenes* in homemade pottery cheese. Tekirdağ Ziraat Fakültesi Dergisi.

[65] Kizanlik P.K., Göksoy E.Ö. (2018). Microbiological quality evaluation of various types of cheese. Erciyes Üniversitesi Vet. Fakültesi Derg..

[66] Şanlıbaba P., Tezel B.U., Çakmak G.A. (2018). Detection of *Listeria* spp. in raw milk and dairy products retailed in Ankara. Gıda.

[67] Şanlıbaba P., Tezel B.U. (2018). Prevalence and characterization of *Listeria* species from raw milk and dairy products from çanakkale province. Turk. J. Agric. Food Sci. Technol..

[68] Albastami I., Wajiej A.H., Aburagaegah S. (2020). Microbiological study on *Listeria* species isolated from some food products of animal origin. Damanhour J. Vet. Sci..

[69] Sobhy M.I., Sayed M., Walaa E. (2022). Influence of essential oils on the viability of *Listeria monocytogenes*. Assiut Vet. Med. J..

[70] Kamat A.S., Nair P.M. (1994). Incidence of *Listeria* species in Indian seafoods and meat. J. Food Saf..

[71] Dominguez C., Gomez I., Zumalacarregui J. (2001). Prevalence and contamination levels of *Listeria monocytogenes* in smoked fish and pate sold in Spain. J. Food Prot..

[72] Hassan Z., Purwati E., Radu S., Rahim R.A., Rahim R.A., Rusul G. (2001). Prevalence of *Listeria* spp and *Listeria monocytogenes* in meat and fermented fish in Malaysia. Southeast Asian J. Trop. Med. Public Health.

[73] Salihu M.D., Junaidu U., Manga S.B., Gulumbe M.L., Magaji A.A., Ahmed A., Adamu A.I., Schittu A., Balarabe I. (2008). Occurrence of *Listeria monocytogenes* in smoked fish in Sokoto, Nigeria. Afr. J. Biotechnol..

[74] Masuda T., Iwaya M., Miura H., Kokubo Y., Maruyama T. (1992). Occurrence of *Listeria* species in fresh seafood. Food Hyg. Saf. Sci..

[75] Bianchini M., Arias M.L., Herrera C., Zuniga C. (1999). *Listeria monocytogenes* incidence and evaluation of the sanitary quality of filleted fresh fish from the Metropolitan Area of San José. Arch. Latinoam. Nutr..

[76] Ripabelli G., Sammarco M.L., Fanelli I., Grasso G.M. (2004). Ricerca di *Salmonella*, *Listeria* spp., *Vibrio* spp. e *Yersinia enterocolitica* in prodotti ittici congelati e surgelati del commercio e correlazione con gli indici di contaminazione fecale: Implicazioni in Sanità Pubblica. Ann. Ig..

[77] Akkaya L., Atabay H.İ., Gök V., Kara R. (2011). Detection of *Listeria* species in fresh fish and fish market environment by IMS technique in Turkey. Archiv fur Lebensmittelhygiene.

[78] Modaresi R., Mardani K., Tukmechi A., Ownagh A. (2011). Prevalence of *Listeria* spp. in fish obtained from Urmia fish markets. Afr. J. Microbiol. Res..

[79] Voidarou C., Alexopoulos A., Plessas S., Noussias H., Stavropoulou E., Fotou K., Tzora A., Skoufos I., Bezirtzoclou E., Demertzi-Akrida K. (2011). Microbiological quality of grey-mullet roe. Anaerobe.

[80] Momtaz H., Yadollahi S. (2013). Molecular characterization of *Listeria monocytogenes* isolated from fresh seafood samples in Iran. Diagn. Pathol..

[81] Abdellrazeq G.S., Kamar A.M., El-Houshy S.M. (2014). Molecular Characterization of *Listeria* Species Isolated from Frozen Fish. Alex. J. Vet. Sci..

[82] Edris A.M., Amany M.S., Michael A.F. (2014). Incidence of *Listeria monocytogenes* in fresh tilapia nilotica fish. Benha Vet. Med. J..

[83] Mashak Z., Banisharif F., Banisharif G., Reza Pourian M., Eskandari S., Seif A., Dehkordi F.S., Alavi I. (2021). Prevalence of *listeria* species and serotyping of *Listeria monocytogenes* bacteria isolated from seafood samples. Egypt. J. Vet. Sci..

[84] Tarazi Y., El-Sukhon S., Al-Rahbi A., Ismail Z.B. (2021). Molecular characterization and in vivo pathogenicity study of *Listeria monocytogenes* isolated from fresh and frozen local and imported fish in Jordan. Open Vet. J..

[85] Chou C.H., Silva J.L., Wang C. (2006). Prevalence and typing of *Listeria monocytogenes* in raw catfish fillets. J. Food Prot..

[86] El-Malek A.M.A., Ali S.F.H., Hassanein R., Mohamed M.A., Elsayh K.I. (2010). Occurrence of *Listeria* species in meat, chicken products and human stools in Assiut city, Egypt with PCR use for rapid identification of *Listeria monocytogenes*. Vet. World.

[87] Osaili T.M., Alaboudi A.R., Nesiar E.A. (2011). Prevalence of *Listeria* spp. and antibiotic susceptibility of *Listeria monocytogenes* isolated from raw chicken and ready-to-eat chicken products in Jordan. Food Control.

[88] Karakolev R. (2009). Incidence of Listeria monocytogenes in beef, pork, raw-dried and raw-smoked sausages in Bulgaria. Food Control.

[89] Sırıken B., Pamuk Ş., Özakın C., Gedikoglu S., Eyigör M. (2006). A note on the incidences of *Salmonella* spp., *Listeria* spp. and *Escherichia coli* O157: H7 serotypes in Turkish sausage (Soudjouck). Meat Sci..

[90] Molla B., Yilma R., Alemayehu D. (2004). *Listeria monocytogenes* and other *Listeria* species in retail meat and milk products in Addis Ababa, Ethiopia. Ethiop. J. Health Dev..

[91] Gebretsadik S., Kassa T., Alemayehu H., Huruy K., Kebede N. (2011). Isolation and characterization of *Listeria monocytogenes* and other *Listeria* species in foods of animal origin in Addis Ababa, Ethiopia. J. Infect. Public Health.

[92] Eruteya O.C., Odunfa S.A., Lahor J. (2014). *Listeria* spp. in raw cow and goat meat in Port Harcourt, Nigeria. Br. Biotechnol. J..

[93] Ismaiel A.A.R., Ali A.E.S., Enan G. (2014). Incidence of *Listeria* in Egyptian meat and dairy samples. Food Sci. Biotechnol..

[94] Ristori C.A., Rowlands R.E.G., Martins C.G., Barbosa M.L., Yoshida J.T., de Melo Franco B.D. (2014). Prevalence and populations of *Listeria monocytogenes* in meat products retailed in Sao Paulo, Brazil. Foodborne Pathog. Dis..

[95] Onyilokwu S.A., Lawan F.A., Hambali I.U., Mailafiya S., Adamu N.B., Atsanda N.N., Jauro S. (2016). Phenotypic Characterisation and Distribution Pattern of *Listeria* Species Isolated from Food Samples Retailed In Markets and Central Abattoir in Maiduguri, Nigeria. Alex. J. Vet. Sci..

[96] Reda W.W., Abdel-Moein K., Hegazi A., Mohamed Y., Abdel-Razik K. (2016). *Listeria monocytogenes*: An emerging food-borne pathogen and its public health implications. J. Infect. Dev. Ctries..

[97] Arslan S., Baytur S. (2019). Prevalence and antimicrobial resistance of *Listeria* species and subtyping and virulence factors of *Listeria monocytogenes* from retail meat. J. Food Saf..

[98] Omogbai B.A., Esokpunwu D.E. (2019). Molecular Characterization and Antibiotic Resistance Patterns of *Listeria* Species in Frozen Beef and Chicken Sold in Benin City, Nigeria. Food Appl. Biosci. J..

[99] Chuku A., Obande G.A., Eya S.B. (2019). Listerial contamination of raw beef and chevon in north-central Nigeria. IMC J. Med. Sci..

[100] Gebremedhin E.Z., Hirpa G., Borana B.M., Sarba E.J., Marami L.M., Tadese N.D., Ambecha H.A. (2021). Detection of *Listeria* species, factors associated, and antibiogram of *Listeria monocytogenes* in beef at abattoirs, butchers, and restaurants of Ambo and Holeta Towns, Ethiopia. Infect. Drug Resist..

[101] Okorie-Kanu O.J., Anyanwu M.U., Ezenduka E.V., Mgbeahuruike A.C., Okorie-Kanu C.O., Ugwuijem E.E., Idogwu M.N., Anyaoha C.O., Majesti-Alugakberie O.L., Vidal R.O. (2020). Occurrence and antibiogram of *Listeria* species in raw pork, beef, and chicken meats marketed in Enugu State, Southeast Nigeria. Vet. World.

[102] Al-Humam N.A., Reda L., Mohamed R.E., El-Ghareeb W.R., Darwish W.S., Ibrahim N.A. (2021). Prevalence and Antibiogram of *Listeria monocytogenes* in Retailed Buffalo Raw Meat and Mince with a Protection Trial Using Nisin, and Gingerol. Buffalo Bull..

[103] Alonso-Hernando A., Prieto M., García-Fernández C., Alonso-Calleja C., Capita R. (2012). Increase over time in the prevalence of multiple antibiotic resistance among isolates of *Listeria monocytogenes* from poultry in Spain. Food Control.

[104] Fallah A.A., Saei-Dehkordi S.S., Rahnama M., Tahmasby H., Mahzounieh M. (2012). Prevalence and antimicrobial resistance patterns of *Listeria* species isolated from poultry products marketed in Iran. Food Control.

[105] Daniel S.T., Umeh E.U., Iheukwumere C.C. (2015). Contamination and antibiotic susceptibility profile of *Listeria* species in frozen and fresh chicken sold in Makurdi, Nigeria. Int. J. Curr. Microbiol. Appl. Sci..

[106] Dahshan H., Merwad A.M.A., Mohamed T.S. (2016). *Listeria* species in broiler poultry farms: Potential public health hazards. J. Microbiol. Biotechnol..

[107] Antoniollo P.C., Bandeira F.D.S., Jantzen M.M., Duval E.H., Silva W.P.D. (2003). Prevalence of *Listeria* spp. in feces and carcasses at a lamb packing plant in Brazil. J. Food Prot..

[108] Pesavento G., Ducci B., Nieri D., Comodo N., Nostro A.L. (2010). Prevalence and antibiotic susceptibility of *Listeria* spp. isolated from raw meat and retail foods. Food Control.

[109] Al-Nabulsi A.A., Osaili T.M., Awad A.A., Olaimat A.N., Shaker R.R., Holley R.A. (2015). Occurrence and antibiotic susceptibility of *Listeria monocytogenes* isolated from raw and processed meat products in Amman, Jordan. CyTA J. Food.

[110] Garedew L., Taddese A., Biru T., Nigatu S., Kebede E., Ejo M., Fikru A., Birhanu T. (2015). Prevalence and antimicrobial susceptibility profile of *Listeria* species from ready-to-eat foods of animal origin in Gondar Town, Ethiopia. BMC Microbiol..

[111] Doijad S.P., Vaidya V., Kalekar S., Rodrigues J., D’Costa D., Boshle S.N., Barbuddhe S.B. (2010). Isolation and characterization of *Listeria* species from raw and processed meats. J. Vet. Pub. Health.

[112] Soriano J.M., Rico H., Molto J.C., Manes J. (2001). *Listeria* species in raw and ready-to-eat foods from restaurants. J. Food Prot..

[113] Conficoni D., Santagiuliana M., Marchesan M., Franceschini F., Catellani P., Ferioli M., Giaccone V. (2019). Distribution of *Listeria* spp. on Carcasses of Regularly Slaughtered Swine for Italian Dry Cured Ham. J. Food Prot..

[114] Rodriguez-Calleja J.M., Garcia-Lopez I., Garcia-Lopez M.L., Santos J.A., Otero A. (2006). Rabbit meat as a source of bacterial foodborne pathogens. J. Food Prot..

[115] El-Gohary A.H., Mohamed A.A., Ramadan H.H., Abuhatab E.A. (2018). Zoonotic and Molecular Aspects of *Listeria* Species Isolated from Some Farm Animals at Dakahlia Province in Egypt. Alex. J. Vet. Sci..

[116] Haggag Y.N., Nossair M.A., Shehab S.A. (2019). Is Raw Milk Still Vehicle for Transmitting *Listeria* species To Pregnant Women?. Alex. J. Vet. Sci..

[117] Vazquez-Salinas C., Rodas-Suarez O., Quinones-Ramirez E.I. (2001). Occurrence of *Listeria* species in raw milk in farms on the outskirts of Mexico City. Food Microbiol..

[118] Omar S.S., Dababneh B.F., Qatatsheh A., Abu-Romman S., Hawari A.D., Aladaileh S. (2011). The incidence of *Listeria* species and other indicator bacteria in some traditional foods sold in Karak city, Jordan. J. Food Agric. Environ..

[119] Yakubu Y., Salihu M.D., Faleke O.O., Abubakar M.B., Junaidu A.U., Magaji A.A., Gulumbe L.M., Aliyu R.M. (2012). Prevalence and antibiotic susceptibility of *Listeria monocytogenes* in raw milk from cattle herds within Sokoto Metropolis, Nigeria. Sokoto J. Vet. Sci..

[120] Al-Mariri A., Younes A., Ramadan L. (2013). Prevalence of *Listeria* spp. in raw milk in Syria. Bulg. J. Vet. Med..

[121] Rahimi E., Momtaz H., Behzadnia A., Baghbadorani Z.T. (2014). Incidence of *Listeria* species in bovine, ovine, caprine, camel and water buffalo milk using cultural method and the PCR assay. Asian Pac. J. Trop. Dis..

[122] Acaröz U., Acaröz-Arslan D., Recep K.A.R.A., Zemheri F., Gürler Z. (2017). Determination of *Listeria* species in water buffalo and cow milk obtained from Afyonkarahisar province. Kocatepe Vet. J..

[123] EL-Naenaeey E.S., Abdelwahab A., Merwad A., Abdou H. (2019). Prevalence of *Listeria* Species in Dairy Cows and Pregnant Women with Reference to Virulotyping of *Listeria monocytogenes* in Egypt. Zagazig Vet. J..

[124] Babacan O. (2021). Determination of the presence and antibiotic resistance of *Listeria* species and aerobic mesophilic bacteria count of cow milks. Veteriner Hekimler Derneği Dergisi.

[125] Rodriguez J.L., Gava P., Medina M., Nuñez M. (1994). Incidence of *Listeria monocytogenes* and other *Listeria* spp. in ewes’ raw milk. J. Food Prot..

[126] Vitas A.I. (2004). Occurrence of *Listeria monocytogenes* in fresh and processed foods in Navarra (Spain). Int. J. Food Microbiol..

[127] Lianou D.T., Skoulakis A., Michael C.M., Katsarou E.I., Chatzopoulos D.C., Solomakos N., Tsilipounidaki K., Florou Z., Cripps P.J., Katsafadou A.I. (2022). Isolation of *Listeria ivanovii* from Bulk-Tank Milk of Sheep and Goat Farms-From Clinical Work to Bioinformatics Studies: Prevalence, Association with Milk Quality, Antibiotic Susceptibility, Predictors, Whole Genome Sequence and Phylogenetic Relationships. Biology.

[128] Gaya P., Saralegui C., Medina M., Nunez M. (1996). Occurrence of *Listeria monocytogenes* and other *Listeria* spp. in raw caprine milk. J. Dairy Sci..

[129] Baher W., Shalaby M., Abdelghfar S. (2021). Prevalence of multidrug-resistant *Listeria monocytogenes* in retailed goat meat and offal. Damanhour J. Vet. Sci..

[130] Hyera E., Msalya G., Karimuribo E.D., Kurwijila L.R., Alonso S., Roesel K., Grace D. Isolation and identification of *Listeria* species along the milk value chain in one region of Tanzania. Proceedings of the First Joint AITVM—STVM Conference.

[131] Usman U.B., Kwaga J.K.P., Kabir J., Olonitola O.S. (2016). Isolation and antimicrobial susceptibility of *Listeria monocytogenes* from raw milk and milk products in Northern Kaduna State, Nigeria. J. Appl. Environ. Microbiol..

[132] El Hag M.M., El Zubeir I.E.M., Mustafa N.E. (2021). Prevalence of *Listeria* species in dairy farms in Khartoum State (Sudan). Food Control.

[133] Borena B.M., Dilgasa L., Gebremedhin E.Z., Sarba E.J., Marami L.M., Kelbesa K.A., Tadese N.D. (2022). *Listeria* Species Occurrence and Associated Risk Factors and Antibiogram of *Listeria monocytogenes* in Milk and Milk Products in Ambo, Holeta, and Bako Towns, Oromia Regional State, Ethiopia. Vet. Med. Int..

[134] Eldaly E.A., Saleh E.A., Moustafa A.H., Atya O. (2013). Prevalence Of *Listeria* Organisms In Meat And Some Meat Products. Zagazig Vet. J..

[135] Aisha B.M., Kawo A.H. (2014). Isolation of *Listeria monocytogenes* recovered from some ready-to-eat foods sold in Kano, North-Western Nigeria. Bayero J. Pure Appl. Sci..

[136] Ndahi M.D., Kwaga J.K.P., Bello M., Kabir J., Umoh V.J., Yakubu S.E., Nok A.J. (2014). Prevalence and antimicrobial susceptibility of *Listeria monocytogenes* and methicillin-resistant *Staphylococcus aureus* strains from raw meat and meat products in Zaria, Nigeria. Lett. Appl. Microbiol..

[137] Mahmoud H., Karmi M., Maky M. (2019). Occurrence and Characterization of *Listeria* Species Isolated from Processed Meat in Qena, Egypt. Zagazig Vet. J..

[138] Nyenje M.E., Odjadjare C.E., Tanih N.F., Green E., Ndip R.N. (2012). Foodborne pathogens recovered from ready-to-eat foods from roadside cafeterias and retail outlets in Alice, Eastern Cape Province, South Africa: Public health implications. Int. J. Environ. Res. Public Health.

[139] Büyükyörük S., Beyaz D., Göksoy E.Ö., Filiz K.Ö.K., Kocak P. (2014). Microbiological evaluation of ready-to-eat sandwiches served near hospitals and schools. Ank. Üniversitesi Vet. Fakültesi Derg..

[140] İşleyici Ö., Sancak Y.C., Tuncay R.M., Atlan M. (2019). Presence of *Listeria* species in ready-made meatballs offered by sale under freezing or cooling preservation. Ank. Univ. Vet. Fakültesi Derg..

[141] Sotohy E.M., Abd EL-Malek A. (2019). Assessment of microbiological quality of ready to eat meat sandwiches in new valley governorate. Int. J. Food Sci. Nutr. Eng..

[142] Kovačević M., Burazin J., Pavlović H., Kopjar M., Piližota V. (2013). Prevalence and level of *Listeria monocytogenes* and other *Listeria* sp. in ready-to-eat minimally processed and refrigerated vegetables. World J. Microbiol. Biotechnol..

[143] Alsheikh A.D.I., Mohammed G.E., Abdalla M.A. (2013). Isolation and identification of *Listeria monocytogenes* from retail broiler chicken ready to eat meat products in Sudan. Int. J. Anim. Vet. Adv..

[144] Ripabelli G., Sammarco M.L., Fanelli I., Grasso G.M. (2002). Prevalenza di *Campylobacter*, *Salmonella*, *Vibrio*, *Yersinia enterocolitica*, *Listeria* ed *Escherichia coli* in vegetali freschi del commercio. L’Igiene Mod..

[145] Francis G.A., O’Beirne D.A.V.I.D. (2006). Isolation and pulsed-field gel electrophoresis typing of *Listeria monocytogenes* from modified atmosphere packaged fresh-cut vegetables collected in Ireland. J. Food Prot..

[146] Stonsaovapak S., Boonyaratanakornkit M. (2010). Prevalence and antimicrobial resistance of *Listeria* species in food products in Bangkok, Thailand. J. Food Saf..

[147] Bouayad L., Hamdi T.M. (2012). Prevalence of *Listeria* spp. in ready to eat foods (RTE) from Algiers (Algeria). Food Control.

[148] Wang F.I., Chern M.K., Li C.W., Yan M., Hsieh Y.H. (2012). Prevalence and antibiotic resistance of *Listeria* species in food products in Taipei, Taiwan. Afr. J. Microbiol. Res..

[149] Ebakota D.O., Abiodun O.A., Nosa O.O. (2018). Prevalence of antibiotics resistant *Listeria monocytogenes* strains in Nigerian ready-to-eat foods. Food Saf..

[150] Camellini S., Iseppi R., Condò C., Messi P. (2021). Ready-to-Eat Sandwiches as Source of Pathogens Endowed with Antibiotic Resistance and Other Virulence Factors. Appl. Sci..

[151] Ramírez Mérida L.G., Morón de Salim A., Alfieri Graterol A.Y., Gamboa O. (2009). Frecuencia de *Listeria monocytogenes* en muestras de tomates (*Lycopersicum esculentum*) y cilantro (*Coriandrum sativum*) frescos en tres supermercados de Valencia, Venezuela. Arch. Latinoam. Nutr..

[152] Mawak J.D., Dashen M.M., Idolo A.J., Chukwu O.O.C. (2009). Occurrence of *Listeria monocytogenes* in irrigation water and vegetable at Jos, Plateau State, Nigeria. Int. J. Trop. Agric. Food Syst..

[153] Hunter R. (1973). Observations on *Listeria monocytogenes* type 5 (Iwanov) isolated in New Zealand. Med. Lab. Technol..

[154] Dennis S.M. (1975). Perinatal lamb mortality in western Australia. Aust. Vet. J..

[155] Hammer Ø., Harper D.A.T., Ryan P.D. (2001). Past: Paleontological statistics software package for education and data analysis. Palaeontol. Electron..

[156] Diriba K., Awulachew E., Diribsa K. (2021). The prevalence of *Listeria* species in different food items of animal and plant origin in Ethiopia: A systematic review and meta-analysis. Eur. J. Med. Res..

[157] Castrica M., Menchetti L., Panseri S., Cami M., Balzaretti C.M. (2021). When Pet Snacks Look Like Children’s Toys! The Potential Role of Pet Snacks in Transmission of Bacterial Zoonotic Pathogens in the Household. Foodborne Pathog. Dis..

[158] U.S. Food and Drug Administration Detection of *Listeria monocytogenes* in foods and environmental samples, and enumeration of *Listeria monocytogenes* in foods. In *Bacteriological Analytical Manual*; 2017; Chapter 10. https://www.fda.gov/food/laboratory-methods-food/bam-detection-and-enumeration-Listeria-monocytogenes.

[159] International Organization for Standardization (2017). Microbiology of Food and Animal Feeding Stuffs—Horizontal Method for the Detection and Enumeration of Listeria Monocytogenes and Listeria spp. ISO 11290-1:2017.

[160] U.S. Department of Agriculture (2019). Food Safety and Inspection Service. Isolation and identification of *Listeria monocytogenes* from red meat, poultry, ready-to-eat Siluriformes (fish) and egg products, and environmental samples, method 8.11. Microbiology Laboratory Guidebook.

[161] Supanivatin P., Kosonpisit S., Liamkaew R., Saeteaw N., Thipayarat A. (2012). Inhibitory effects of *Listeria* selective enrichment media on growth characteristics of *L. ivanovii*. Procedia Eng..

[162] Liu D., Ainsworth A.J., Austin F.W., Lawrence M.L. (2004). PCR detection of a putative N-acetylmuramidase gene from *Listeria ivanovii* facilitates its rapid identification. Vet. Microbiol..

[163] Liu D., Lawrence M.L., Austin F.W., Ainsworth A.J. (2007). A multiplex PCR for species- and virulence-specific determination of *Listeria monocytogenes*. J. Microbiol. Methods.

[164] Li F., Ye Q., Chen M., Zhang J., Xue L., Wang J., Wu S., Zeng H., Gu Q., Zhang Y. (2021). Multiplex PCR for the Identification of Pathogenic *Listeria* in *Flammulina velutipes* Plant Based on Novel Specific Targets Revealed by Pan-Genome Analysis. Front. Microbiol..

[165] Mao Y., Huang X., Xiong S., Xu H., Aguilar Z.P., Xiong Y. (2016). Large-volume immunomagnetic separation combined with multiplex PCR assay for simultaneous detection of *Listeria monocytogenes* and *Listeria ivanovii* in lettuce. Food Control.

[166] Xiao F., Bai X., Wang K., Sun Y., Xu H. (2022). Rapid-Response Magnetic Enrichment Strategy for Significantly Improving Sensitivity of Multiplex PCR Analysis of Pathogenic *Listeria* Species. Appl. Sci..

[167] Rodríguez-Lázaro D., López-Enríquez L., Hernández M. (2010). *smc*L as a novel diagnostic marker for quantitative detection of *Listeria ivanovii* in biological samples. J. Appl. Microbiol..

[168] Wang Y., Wang Y., Xu H., Dai H., Meng S., Ye C. (2014). Rapid and sensitive detection of *Listeria ivanovii* by loop-mediated isothermal amplification of the *smcL* gene. PLoS ONE.

[169] Barbuddhe S.B., Maier T., Schwarz G., Kostrzewa M., Domann E., Chakraborty T., Hain T. (2008). Rapid identification and typing of *Listeria* species using matrix assisted laser desorption ionization-time of flight mass spectrometry. Appl. Environ. Microbiol..

