# Supplementary material for: Virulence Characteristics and Distribution of the Pathogen Listeria ivanovii in the Environment and in Food
Source: Microorganisms. 2022 Aug 20;10(8):1679. doi: 10.3390/microorganisms10081679 (PMC9414773; doi:10.3390/microorganisms10081679)
Supplement: Supplementary file 1 [file microorganisms-10-01679-s001.zip › microorganisms-1869330-supplementary.pdf]

Table S1. Reports of *L. ivanovii* isolation from food products in chronological order with type of food, total number of samples tested in the same study, country, analytical methods used and reference.

Food	Total samples tested	Country	Analytical methods[1]	Number and (%) of samples contaminated with *L. ivanovii*	*L. ivanovii* positive food samples and number	Reference
Commercial fresh seafood (retail)	683	Japan	b	2 (0.3)	Fish	[74]
Fresh, frozen and dry seafoods (retail)	38	India	b (different methods)	6 (15.8)	Local fish doma (2); crab (2); dry fish shrimp (2)	[70]
Fresh meat and meat products (retail)	27	India	b (different methods)	8 (29.6)	Pork cocktail (2); chicken sausages (2); pork salami (2); raw chicken (2)	[70]
Raw ewe milk	1,052	Spain	b	2 (0.2)		[121]
Raw goat milk (farm)	1445	Spain	a3, c1	3 (0.2)		[124]
Fresh filleted fish	135	Costa Rica	a2	3 (2.2)	Filets de bar and dorate	[75]
Ready to eat (retail) and raw food	429	Portugal	b	6 (1,6)	Cheese (3); RTE meat (1); raw milk (2)	[52]
Smoked fish and pâté	352	Spain	a2	2 (0.6)	Opened cold-smoked salmon (1); vacuum-packed cold-smoked trout (1)	[71]
Ready to eat and raw food in restaurant	103	Spain	a3	6 (5.8)	lettuce (3); RTE lettuce (1); raw pork (2)	[109]
Local and imported meat and fermented fish (retail)	101	Malaysia	b,	36 (35.6)	Imported frozen beef (14); local frozen meat (9); fermented Kedah fish (13)	[72]
Raw cow milk	1300	Mexico	a3	82 (6.3)		[113]
Fresh vegetables (retail)	188	Italy	a2	1 (0.5)	lettuce	[144]
Raw ewes milk	542	Spain	a3	5 (0.9)		[122]
Swabs on lamb carcass surfaces (abattoir)	69	Brazil	a2	1 (1.4)		[104]
Meat and milk products (retail)	316	Ethiopia	a1.1	1 (0.3)	Minced beef	[91]
Frozen seafood (retail)	213	Italy	a2	5 (2.3)	Mussels (3); cam (1); shrimp (1)	[76]
Raw milk (dairy plants), cheese, yoghurt, butter (retail)	157	Turkey	a3	4 (2.5)	Raw milk (1); Turkish white cheese (3)	[53]
Catfish fillets	240	USA	a3, c	2 (0.8)		[85]

RTE fresh-cut fruits and vegetables in MAP (retail)	720	Ireland	a2, c	1 (0.1)	Bean sprouts	[145]
Rabbit carcasses (abattoir) and rabbit raw meat (retail)	51	Spain	a3, IMS, c	1 (2.0)		[108]
RTE Turkish sausage (Soudjouck)	100	Turkey	A3	1 (1.0)		[90]
Animal food products (street markets)	1481	Colombia	b, c	32 (2.2)	Cheeses type “costeño” (32)	[54]
Homemade white cheeses (bazaar)	142	Turkey	a3	3 (2.1)		[55]
Bulk untreated sheep, goat and sheep-goat milk (farms)	246	Italy	a1.1, c	1 (0.4)		[56]
Sheep-goat cheese (dairy plants)	71	Italy	a1.1, c	2 (2.8)		[56]
Smoked fish (retail)	115	Nigeria	a1.1	15 (13)		[73]
Various raw and RTE meat	786	Bulgaria	a1.1	5 (0.6)	Minced beef (2) and pork (1); raw-dried sausage (2)	[89]
Raw and RTE foods (retail)	711	Ethiopia	a1.1	4 (0.6)	Minced beef (2); soft cheese (2)	[57]
Vegetables leafy collected	10	Nigeria	b	1		[152]
Fresh vegetables (retail)	192	Venezuela	a7	48 (25.0)	Tomato (16); coriander (32)	[151]
Meat and chicken products	100	Egypt	a3, c	7 (7)	Luncheon (1); frozen chicken fillet (3); frozen chicken leg (3)	[86]
Raw meat and RTE products (schools’ refectories, restaurant, etc.)	1268	Italy	a1.1	10 (0.8)	Only raw meat (no. 575 total samples) 1.7%	[105]
Raw and RTE foods (retail)	380	Thailand	a1.1	3 (0.8)		[146]
Raw and processed meats	109	India	a2, c	1 (0.9)		[127]
White cheeses	30	Venezuela	a3, c	3 (10.0)		[59]
Fresh fish (retail)	100	Turkey	a2+IMS	1 (1)	Trout	[77]
Village cheeses without starter cultures (bazaars)	58	Turkey	a1.1	1 (1.7)		[58]
Raw and RTE foods (retail, cafeterias)	391	Ethiopia	a3	2 (0.5)	Raw beef and liquid whole egg	[92]
Raw milk	250	Turkey	a3	1 (0.4)		[60]
Herby cheese	254	Turkey	a3	1 (0.4)		[60]

Fresh whole fish (retail)	194	Iran	b, c	7 (3.6)		[78]
Traditional RTE foods	100	Jordan	a3	20 (20.0)	Dairy product (8); other RTE (12)	[114]
Raw cow's milk	20	Jordan	a3	7 (35)		[114]
Fresh chicken-neck skin (abattoir)	160	Jordan	a1.1, c	48 (30.0)		[87]
RTE chicken products (retail, restaurant)	120	Jordan	a1.1, c	25 (20.8)	Chicken-shawirma (20); chicken-burger (3); chicken-sausage (2)	[87]
Fish grey-mullet roe (*Mugil cephalus*)	48	Greece	a2	3 (6.3)		[79]
Raw poultry (retail)	100	Spain	a2	1 (1.0)		[88]
RTE foods (various places)	227	Algeria	a4	3 (1.3)		[147]
Raw and RTE poultry products (retail, restaurant)	402	Iran	a2, c	13 (3.2)	Poultry products: raw (10); RTE (3)	[131]
RTE foods	252	South Africa	b	51 (20.2)	Chicken stew (11); beef stew (6); vegetables (8); rice (6), potatoes (6); pies (14)	[138]
Raw and RTE various food (local retail)	400	Taiwan	a1.1	4 (1)	Not specified	[148]
Cheeses and dairy desserts (retail)	300	Turkey	a3	2 (0.7)	Mihalic cheese (2)	[61]
Brined white cheese (retail)	350	Jordan	a1.1, c	14 (4.0)		[62]
Raw cow's milk (farm)	192	Nigeria	a1.1	8 (4.2)		[115]
Raw milk	766	Syria	a3, c	12 (1.6)	Cow milk (7); sheep milk (5)	[116]
Broiler chicken RTE (restaurant)	250	Sudan	a1.1	52 (20.8)	Frozen chicken burger (14); frozen chicken sausages (4); frozen chicken meat balls (kofta) (15); chicken shawarma (6); chicken mortadella (13)	[143]

Soft cheese, raw milk, red and chicken meat (retail)	225	Iraq	a3, c	1 (0.4)	Soft cheese	[63]
Raw and RTE meat	250	Egypt	b, c	1 (0.4)	Raw meat kofta	[134]
RTE vegetables (retail)	100	Croatia	a1.1	5 (5.0)	Iceberg lettuces (3); delicatessen salads (1); shredded cabbages (1)	[142]
Raw fish (retail)	300	Iran	a1.1, c	3 (1)	Fresh fish	[80]
RTE foods (retail)	336	Nigeria	a3	31 (9.2)	Lettuce (10); cabbage (9); meat pie (4); other RTE meat (8)	[135]
Frozen raw fish (imported)	219	Egypt	a1.1, c	2 (0.9)	Flesh catfish	[81]
RTE sandwiches (street vendors)	270	Turkey	a1.1	5 (1.9)	With cheese (1); with soudjouk (dried beef sausage) and salad (3); with soudjouk without salad (1)	[139]
Fresh Tilapia nilotica (retail and farm)	100	Egypt	a3, c	8 (8.0)	Samples in retail (6); samples in farm (2)	[82]
Raw cow and goat meat (retail)	240	Nigeria	a2, c	4 (1.7)	Cow flesh (2); cow kidney (1) and liver (1)	[93]
Raw and RTE food products of animal origin (retail)	180	Egypt	a2	4 (2.2)	Raw lean beef (1); frozen lean beef (2); raw milk (1)	[94]
Raw meat and meat products (retail)	300	Nigeria	b, c	19 (6.3)	Raw meat (5); meat products (14, of which no. 10 of Suya, a smoked spiced meat)	[136]
Raw various animal milk	260	Iran	a2, c	2 (0.8)	Bovine milk (1); ovine milk (1)	[117]
Meat products (retail)	552	Brazil	a1, c	1 (0.2)	Chicken leg	[95]
Raw meat and RTE meat (retail)	270	Jordan	a1.1, c	73 (27.0)	Raw meat (19)<br>RTE meat (54)	[106]
Various food	432	Republic of Ireland	a1.1, c	1 (0.2)	Meat sausage	[28]

Raw frozen and fresh chicken meat	120	Nigeria	b	2 (1.7)	Frozen (1) and fresh (1) meat	[132]
Raw and RTE foods of animal origin (retail, restaurant)	384	Ethiopia	a1	2 (0.5)	Raw meat (2)	[107]
Raw milk, dairy and meat products (retail markets and grocery stores)	233	Egypt	a3	5 (2.2)	Raw milk (2); Kareish cheese (1); butter (1); burger (1)	[50]
Raw chicken meat (farm)	80	Egypt	a1, c	9 (11,3)		[133]
Raw zebu milk	103	Tanzania	a1.1	2 (1.9)		[126]
Homemade cheeses (village bazaars)	279	Turkey	a1.1	6 (2.2)		[64]
Raw meat and vegetables (retail)	330	Nigeria	a3	44 (13.3)	Red meat (17); chicken (6); vegetable (21)	[96]
Raw milk and meat (retail)	250	Egypt		2 (0.8)	Beef burger (1); sausage (1)	[97]
Raw milk and milk products	550	Nigeria	b, c	27 (4.9)	Not specified	[128]
Raw milk	200	Turkey	a1.1, c	1 (0.5)	Cow milk	[118]
Raw milk, cheese, butter (retail)	300	Turkey	a3, c	8 (2.7)	Raw milk (5); white cheese (2); butter (1)	[51]
RTE foods (retail)	411	Nigeria	a1.2	6 (1.5)		[149]

Raw milk (dairy animals)	60	Egypt	b, c	3 (5.0)	cow (1); buffalo (1); ewe (1)	[111]
Cheeses (processing plant output shops)	120	Turkey	a1.1	6 (5.0)		[65]
Raw and pasteurized milk and cheeses	110	Turkey	a1.1	6 (5.5)	Raw milk (1); cheese (5)	[66]
Raw and pasteurized milk and cheeses	200	Turkey	a1.1, c	7 (3.5)	Raw milk (3); white cheese (1); homemade cheese (3)	[67]
Raw ground beef, and chicken meat (retail)	124	Turkey	a1.1	5 (4.0)	Ground beef (5)	[98]
Pig carcasses	150	Italy		17(11.3)		[110]
Raw cow's milk (dairy farms)	200	Egypt	b, c	12 (6)		[119]
Milk (private farms and retail)	250	Egypt	b, c	5 (2)	Cow milk (2); Buffalo milk (2); ewe milk (1)	[112]
Refrigerated and frozen RTE meatballs (various Turkish type) (retail)	290	Turkey	a2, c	9 (3.1)	Frozen turkey meatballs (1); refrigerated (7) and frozen (1) beef meatballs	[140]
Raw meat and meat products (retail)	120	Egypt	a1.1, c	12 (10)	Minced meat (2); Kofta (1); sausage (2); burger (3); luncheon (2); Pasterma (2)	[137]
Frozen raw beef and chicken meat	240	Nigeria	a3, c	12 (5.0)	Beef meat (7); chicken meat (5)	[99]

RTE meat sandwiches (retail)	120	Egypt	b, c	1 (0.8)	Beefburger	[141]
Raw and RTE foods (retail)	200	Libya	a1.1, c	15 (7.5)	Raw milk (7); kariesh cheese (2); beef meat (1); hot dog (2); Tilapia fish (3)	[68]
Raw meat	104	Nigeria	a1.1	22 (21.2)	Beef meat (12); Chevon meat (10)	[100]
Raw meat (abattoirs, retail, restaurants)	450	Ethiopia	a1.1	10 (2.2)		[101]
Raw meat (retail)	432	Nigeria	a1.1, c	7 (1.6)	Chicken meat (3); beef meat (4)	[102]
Raw buffalo meat	100	Egypt	a6	1 (1)		[103]
Raw cow milk (farm)	68	Turkey	a1.1	3 (4.4)		[120]
Raw goat meat and offal (retail)	20	Egypt	a6, c	4 (20.0)	Liver (1); kidney (1); rumen (2)	[125]
Sandwiches in MAP (automatic distributors and retail)	24	Italy	b	3 (12.5)		[150]
Raw milk (dairy farms)	120	Sudan	a1.1	2 (1.7)		[129]
Fresh seafood	350	Iran	a1.1, c	8 (2.3)	Fish (3); shrimp (2); lobster (2); crab (1)	[83]

Fresh and frozen fish (gills and muscle)	510	Jordan	b, c	21 (4.1)	Fresh fish (16); frozen fish (5)	[84]
Raw milk (dairy farm) and dairy products (retail)	482	Ethiopia	a1.1	6 (1.2)		[130]
Raw ewe milk (farms)	325	Greece	a1.1, c	3 (0.9)		[123]
Soft and hard cheeses (retail)	225	Egypt	a3, c	48 (21.3)	Soft cheese (34); artisan hard cheeses (14)	[69]

[1]Test methods used: a1.1, ISO 11290-1, a1.2, ISO 11290-2; a1, both of them; a2, USDA; a3, FDA; a4, AOAC; a5, AFNOR; a6, other; a7, MPN enumeration; b, non-standard test method (e. g. internal laboratory methods and modified standard procedures); c, PCR confirmation of *Listeria* spp. and identification of *L. monocytogenes* serotypes; c1, DNA hybridization test.

IMS: Immunomagnetic separation.

RTE: ready to eat
